# Sources of contamination in sediments of retention tanks and the influence of precipitation type on the size of pollution load

**DOI:** 10.1038/s41598-023-35568-9

**Published:** 2023-06-01

**Authors:** Karolina Matej-Łukowicz, Ewa Wojciechowska, Tomasz Kolerski, Nicole Nawrot, Karol Kuliński, Aleksandra Winogradow

**Affiliations:** 1 Faculty of Civil and Environmental Engineering, Gdańsk University of Technology, Narutowicza 11/12, 80-233 Gdańsk, Poland; 2 Institute of Oceanology of the Polish Academy of Sciences, Powstańców Warszawy 55, 81-712 Sopot, Poland

**Keywords:** Environmental chemistry, Environmental impact, Environmental sciences, Hydrology, Natural hazards

## Abstract

Densification of cities and urban population contributes to increased runoff and suspended solids and alteration of the urban water cycle. Nowadays, Blue-Green Infrastructure is promoted to increase a city’s resilience to floods; however, stormwater drainage systems, supported with retention tanks are still important in protecting urban areas against floods. Sediment accumulation in stormwater infrastructure relates to an issue of pollutants such as heavy metals, nutrients etc. Research on the origin of the pollutants associated with the suspension and ultimately sediment accumulated in sewage can bring new insights about processes in urban catchment areas. This is the first study, which is focused on the analysis of stable carbon and nitrogen isotopes in bottom sediments collected from municipal retention tanks to verify the origin of the deposited pollutants immediately after pluvial floods. The research was additionally extended with water quality analyzes immediately after three types of weather: a dry period, typical precipitation (< 30 mm) and torrential rainfalls (2 events with daily precipitation over 30 mm which caused pluvial flooding of the city area). Analyses of sediments indicated that the main source of carbon and nitrogen in the bottom of the retention tanks had been brought with stormwater runoff from the city area. Organic nitrogen fertilizers appeared to be the main source of nitrogen, while the sources of organic carbon were mixed: C3 land plants, wood, and oil. Additionally, it was found that torrential rainfall caused a 23-fold increase of N-NO_3_ concentration, a sevenfold increase of P-PO_4_ concentration, and an over fivefold increase of concentration of organic matter, in comparison to typical precipitation.

## Introduction

Urbanization contributes to an increase in the amount of suspended solids flowing into the waters. The result is an additional supply of nutrients, organic matter, pesticides and other pollutants which causes degradation of the freshwater systems around the world. The inflow of suspended solids also contributes to increased turbidity, reduces the penetration of light into deeper layers of watercourses and reservoirs, as well as affecting the morphology of the channels and water infrastructure. According to Walling and Collins^1^, transport of nutrients with sediment and contamination by suspension are the greatest threats to freshwater, because it is a vector for other pollutants (heavy metals, microplastics, pharmaceuticals). Moreover, most climate change scenarios predict an irreversible increase in soil erosion, accompanied by a relevant change in rainfall patterns^2^.

Solutions that protect urban areas against floods are divided into retention and infiltration and are called Green Infrastructure^3^. Nowadays, the most effective way of stormwater management and city protection against pluvial floods is a multi-level protection. The first level is a blue and green infrastructure strategy (such as rain gardens, green roofs etc.), contributing to the limitation of runoff and possible accumulation of water in the catchment to facilitate the infiltration or reuse of stormwater at the place of origin. According to this conception, retention tanks represent the second level of protection. In addition to flood protection in the event of short-term torrential rainfall, they make it possible to provide an alternative source of water to improve the reliability and security of water supply (including treatment processes)^4,5^, and serve as recreational areas; thus, they can provide various ecosystem services, depending on their size, location and societal needs.

A key limitation of retention tanks, with regard to flood protection, is the fact that they have a fairly limited effect on peak flows during prolonged rainfall. This limitation occurs when the tanks fill up completely during the rainfall and do not take over the inflow during further rainfall. On such occasions, they additionally become prone to failures, resulting in an uncontrolled outflow of floodwater. Hydrological analyzes, mainly aimed at calculating reservoir retention capacity and filling control, have already been extensively described in the literature. These works were based either on one-dimensional SWMM model^6–8^ and two-dimensional CADDIES^9^, or a more extensive SWAT model^10^, which have already had satisfactory results in forecasting threats^11,12^. Most of the research focusing on reservoirs, however, relates only to water retention, forgetting about pollutants transported by rainwater. The incoming water contains suspended solids which are a vector for, inter alia, heavy metals, phosphorus compounds, and PAHs, which sink to the bottom of the tanks, causing the accumulation of solids^13,14^. Numerous processes take place at the sediment water boundary, including sedimentation, re-suspension and deposition of bottom sediments, which were discussed in detail by Lu et al.^15^ and Nawrot et al.^16^. A number of studies referred to sediment and/or water quality analyses of urban watercourses and retention reservoirs^16–19^. Although the dominant process is sedimentation (during normal flow conditions), even so pollutants deposited into sediments can pose a risk of re-contamination when resuspension takes place, for instance during flood episodes^20,21^. Each of these aspects was described separately; however, comprehensive studies covering all the above aspects are lacking. Amundson et al.^22^ noticed that changes in land use and anthropopressure contribute to an increase in erosion processes, and thus to an increase in suspended solids loads. The search for methods to verify the source of contaminats accumulated in bottom sediments has been carried out using several sediment fingerprinting methods. For many years, methods related to geochemical research or fallout radionuclides research—and even a combination of the two—were widely used. However, these methods lack reference to the analysed area. In contrast, isotope analysis is a useful and precise tool, which points to the origin of selected elements. In the isotope analysis, the characteristic values refer to C3 and C4 plants (these are groups of plants depending on the course of photosynthesis) as well as to local soils. The values close to those characteristic ones precisely indicate the elemental origin.

The main aim of our study was to analyze the stable isotopes of nitrogen and carbon deposited in the bottom sediments of retention tanks and to track the origin of nitrogen and organic matter gathered in the sediments. Another objective was to research the impact of torrential rainfall resulting in pluvial floods, as a potential source of the the most significant pollutant loads, on the water quality and volume of pollutants (N-NO_2_, N-NO_3_, N-NH_4_, P-PO_4_, Ptot, COD, and TSS) carried along to the sea. The study covered two years (2016–2017), during which two torrential rainfall incidents occurred in the summer seasons (July 2016 and July 2017).

## Methodology

The sources of pollution in an urban catchment area are similar in many places, but the amount and proportion of pollutants may vary. The findings of our study contribute to understanding of the role of retention tanks in the city, not only in flood prevention but also in terms of pollutant trapping and removal. The samples were collected on the Oliwski Stream in Gdańsk, northern Poland. The stream outflows directly to the Baltic Sea. It is one of the longest streams in the city, with as many as 13 reservoirs for protection against floods located on it.

Two types of samples were collected during this study: bottom sediment samples and water samples. Sediment samples from four retention tanks were collected as a marker and to obtain information about the sources of pollution from a long period of sedimentation. In the summer seasons of 2016 and 2017, two torrential rainfalls occurred in the analysed catchment. Such high precipitation events had not been noted in this area over the past 100 years of meteo observations. The rainfall episode in 2016 was classified as a 600-year rainfall. Therefore, the data on water quality in the stream were divided into three sets: the period without precipitation (dry weather), after normal rainfall (wet weather) and after torrenatial rainfall. The water pollution of an urban stream outflowing to the sea was compared with these two pluvial flood episodes, for situations occurring during dry weather (precipitation below 5 mm) as well as for rainfall between 5 and 30 mm.

- The third data set is supported with information on rainfall height, duration and intensity from a local observation station and data on the water level in retention tanks on Oliwski Stream. Based on the measurements, the HCMS model was made to calculate the water flow rate, which was used to determine the pollutant loads discharged by the Oliwski Stream into the sea.

### Study area

**Figure 1:**
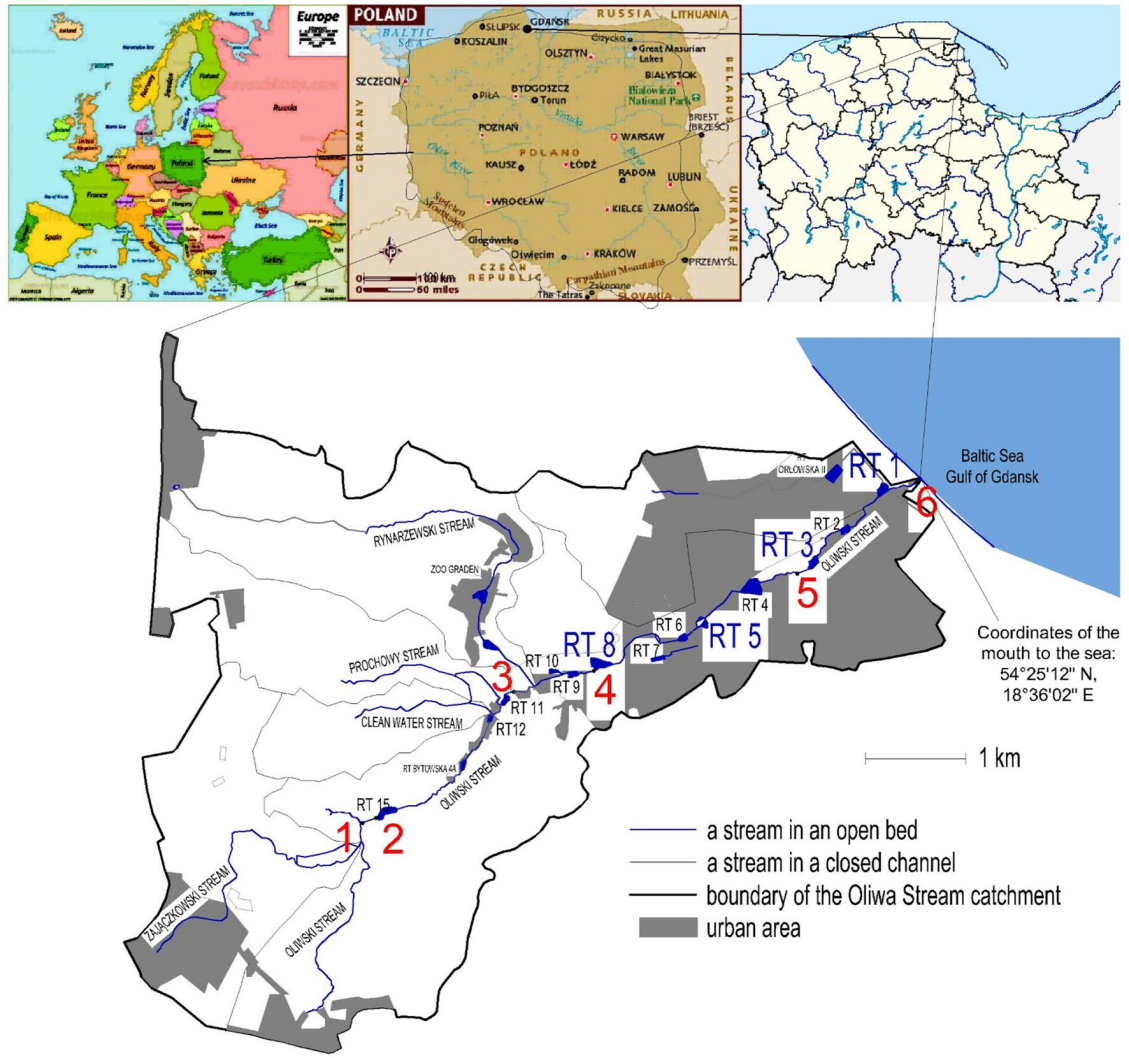
Map with the location of sampling points along Oliwski Stream. Red numbers 1–6 indicate the water sampling points. The red numbers RT8, RT5, RT3, and RT1 indicate the sediment sampling points (RT—retention tank). The map was drawn in AutoCad based on google maps.

Gdańsk is a city with an area of over 260 km^2^, with a population of 464,000 people in 2017, and 471,000 people in 2020. Stormwater from the city outflows to the Baltic Sea by an urban drainage system or via streams. One of the watercourses collecting stormwater and flowing directly to the Gulf of Gdańsk is the Oliwski stream, whose catchment area is equal to 28.92 km^2^ (almost 43% urbanized), and its length is almost 10 km. Along the stream, 13 retention tanks were built, occupying an area of 13.5 hectares and collecting over 70,000 m^3^ of water (Fig. 1).

The Oliwski basin is a typical well-defined channel, which consists of a network of various quarries, forming perennial streams. Since the middle of thefifteenth century, the streams’ energy has been utilized as a resource for a number of watermills together with reservoirs. At present, these hydraulic structures are regarded as historical objects, and reservoirs are used as flood control storage. In general, spillage from the reservoirs are not controlled against flooding. Therefore, following a flood situation, the reservoirs have little impact on surge attenuation and delaying the peaked discharge. The capacity storages of the reservoirs have been diminished by sediment deposition and vegetation distribution.

**Figure 2:**
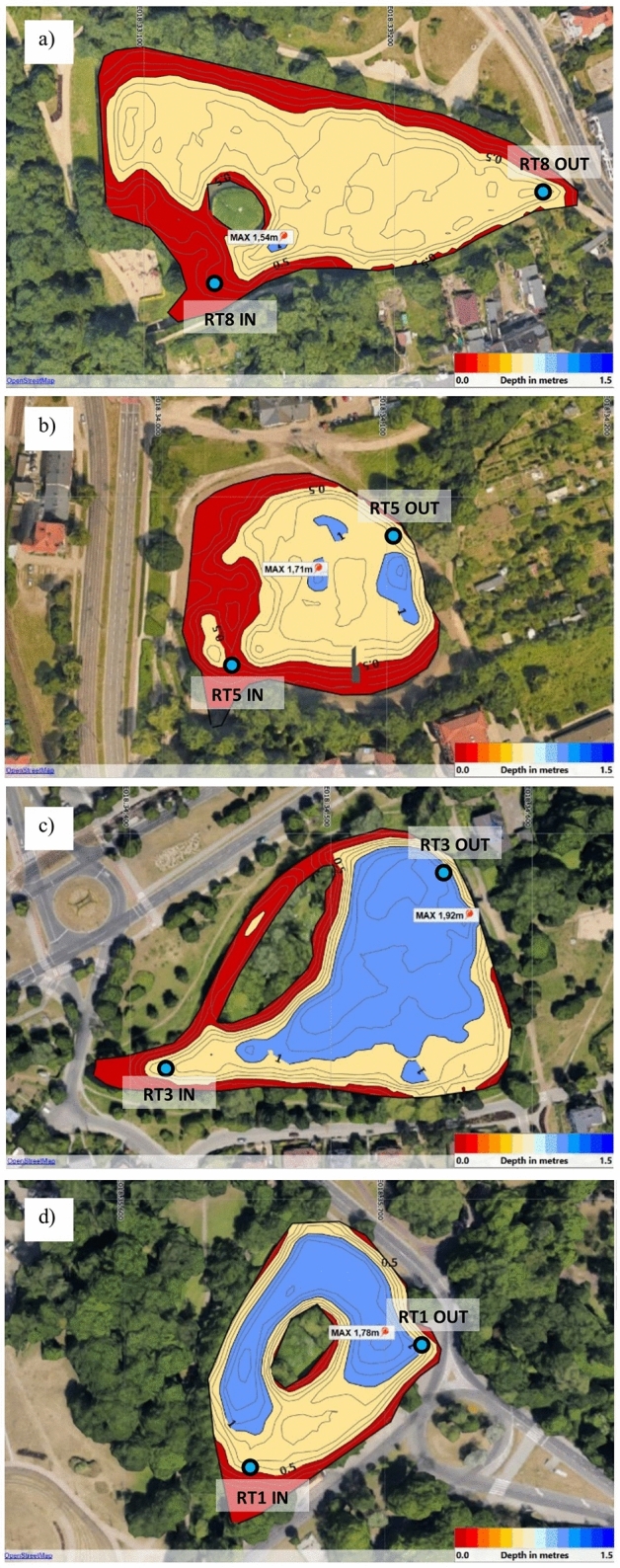
Bathymetric maps of selected retention reservoirs with sampling points (**a**) RT 8, (**b**) RT 5, (**c**) RT 3, (**d**) RT 1^23^. The map was drawn in OpenStreetMap based on google maps.

Figure 2 presents the bathymetry of four analysed retention tanks. These four RTs were selected due to their location in the urbanized part of the catchment area. The depth of the analysed tanks does not exceed 2.0 m. RT 8 (named Spacerowa) is the shallowest tank; on most of its surface, the depth is about 1.0 m. It is located behind the forest catchment, very close to a busy street. The area of RT8 is 10,800 m^2^ and the volume is 5,040 m^3^. RT 5 (named Grunwaldzka) is located behind the city park, on the main street in Gdańsk. It is slightly deeper than the RT8 tank. The area of RT5 is 16,900 m^2^ and the volume is 8,450 m^3^. The RT3 (named Chlopska) is located between residential neighborhoods, also close to the road, but with less traffic. It is the deepest tank; most of the tank's surface is deeper than 1 m. The area of RT3 is 12,000 m^2^, and the volume is 6000 m^3^. The last RT1 (called Jelitkowska), is located closest to the mouth of the stream to the sea, in the tourist part of the city, near the beach, next to a road with much less traffic. This tank has a large island with vegetation, which is the habitat of birds. Most of it is more than 1.0 m deep. The area of RT1 is 10,100 m^2^ and the volume is 5,050 m^3^.

b. Sampling
i. Bottom sediments

Sediment samples were collected from 8 points from 4 retention tanks (RTs), one at the inlet and one outlet of each pond. Eight sampling points were located on four retention tanks (RT1, RT3, RT5 and RT8), two sampling points on each RT (Fig. 1).

Samples of bottom sediment cores were collected during the summer of 2017, using a core sampler consisting of a probe made of acrylic glass, which was hammered at selected points in the bottom of reservoirs. Before removing the probe with sediments, access to air was cut off to protect the sediment layers from displacement. The probe was then placed on a special tripod that was used to eject bottom sediment that resulted from the gradual lowering and ejection of contents through a special internal pin. This sampling method made it possible to divide the sample into 5 cm thick layers. The depth of the extracted core depended on the amount of deposited bottom sediments. Cores with a depth of 0.60 m were extracted from the RT1 from both points (IN and OUT). From the RT3 from the IN and OUT points, 0.55 and 0.70 m respectively, from RT5, 0.50 and 0.45 m, from the RT8 0.60 and 0.65 m. The sediment samples from the successive layers were placed in disposable string bags, frozen, and stored at − 20 °C until analysis. A total of 93 sediment samples were analysed.

ii. Water samples from Oliwski Stream

Water samples were collected from 6 points along the Oliwski Stream (marked in red on Fig. 1). The samples were collected, in the period from June 2016 to September 2017, during three types of weather: (a) a dry period without precipitation (< 5 mm), (b) after "typical" rainfall (wet weather) (5–30 mm), and (c) after torrential rainfall (> 30 mm). Altogether, 11 sampling events were collected during dry weather and 11 sampling events immediately after typical rainfall, with frequency approx. once per month. During the investigation period, two extensively torrential rainfalls occurred on 16th July 2016 and 27th July 2017; the samples were scoop in the morning on the following days.

Water samples for physico-chemical analysis were collected with a scoop or directly from the middle part of the stream to bottles (plastic or glass), with a volume of 1 L. During rain-free periods, samples were collected over a 1–2-h period with a frequency of 10–15 min, with the final sample being a composite sample. The samples were transported without preservation in a portable refrigerator to the laboratory to carry out the analysis within 4 h after collection.

c. Laboratory analyses
i. Bottom sediments

Frozen samples of bottom sediments were prepared for analysis of organic carbon (Corg), total nitrogen (Ntot), stable isotopes of carbon (δ^13^C), and nitrogen (δ^15^N). At the first step, samples were thawed at room temperature, then transferred to Petri dishes, previously weighed and described. The samples in the dishes were placed in a laboratory dryer at a temperature of 60 °C and dried to constant weight. Then, samples were homogenized using an agate mortar, previously removing ingredients that could not be ground (e.g., branches, solid metal waste, fabric, and plastic fragments).

Corg, Ntot, δ^13^C, and δ^15^N concentrations were measured in an Elemental Analyzer Flash EA 1112 Series, combined with the Isotopic Ratio Mass Spectrometer IRMS Delta V Advantage (Thermo Electron Corp., Germany), using high-temperature combustion (oxidation at 1020 °C, followed by reduction over copper at 680 °C). To remove carbonates, dry and homogeneous samples of the sediments were weighed into silver capsules and acidified with 2 M HCl. Quality control included measurements of blanks and certified reference materials (LKSD-1, “flußsediment”), provided by HEKAtech GmbH (Germany). The analyses provided satisfactory accuracy and precision (average recovery 97.1 ± 2.0%, precision given relative to SD was 1.5%). Isotopic ratios δ^13^C and δ^15^N were calculated using pure reference gases: CO_2_ and N_2_ calibrated against IAEA standards: CO-8 for δ^13^C and N-1 for δ^15^N. The results of δ^13^C and δ^15^N are given in the conventional delta notation, i.e., versus PDB for δ^13^C and versus air for δ^15^N according to Eq. (1).

1 $$\frac{{\delta }^{13}C}{{\delta }^{15}N}=\left[\left(\frac{{R}_{sample}}{{R}_{standard}}\right)-1\right]\times 1000$$

where R is the 13C/12C and 15N/14N ratios.

The ratio of carbon to total nitrogen was determined using formula (2).

2 $$C/N=\frac{Corg [\%]/12}{Ntot [\%]/14}$$

where $Corg [\mathrm{\%}]$—the percentage of organic carbon in the sediment sample, $Ntot [\%]$*—*the percentage of total nitrogen in the sediment sample.

ii. Water

The concentrations of nitrogen (N–NO_2_, N–NO_3_, N–NH_4_, Ntot), phosphorus (P–PO_4_, Ptot), and COD were investigated using Hach Lange cuvette tests immediately after the samples were delivered to the laboratory^24–27^. The Hach VIS DR3900 spectrophotometer was used for the measurements. Mineralization of organic matter to inorganic products was done in a Hach HT200S high-temperature thermostat. All measurements were performed in triplicate, and the result was calculated as the arithmetic mean of the three repetitions.

d. Meteo data and rainfall events

The amount of precipitation was measured at the weather station located in the catchment area of Oliwski Stream (150 m from point no 3 (Fig. 1)). Measurements were made every minute with an accuracy of 0.1 mm and transmitted to a remote reading system. Classification of three kinds of rainfall (in three types of weather) and dates is presented in the Table 1.

**Table 1:** Precipitation height and rainfall event classification in 13 rainfall episodes.

No	Rain-free period	After rainfall	Precipitation height (mm)	Precipitation category
1	12-May-16	2-Jun-16	30.0	Typical
2	–	15-Jul-16	178.1	Torrential
3	20-Aug-16	22-Aug-16	13.9	Typical
4	18-Oct-16	21-Oct-16	19.7	Typical
5	29-Nov-16	2-Dec-16	25.8	Typical
6	24-Jan-17	21-Feb-17	10.6	Typical
7	16-Mar-17	19-Mar-17	7.7	Typical
8	2-Jun-17	7-Jun-17	23.5	Typical
9	28-Jun-17	1-Jul-17	14.0	Typical
10	–	27-Jul-17	114.3	Torrential
11	26-Aug-17	27-Aug-17	25.6	Typical
12	7-Nov-17	12-Nov-17	27.8	Typical
13	1-Mar-18	3-Mar-18	7.1	Typical

The key criterion of typical rainfall was exceeding 5 mm in height, because such rainfall caused an inflow to the sewage network and receivers. The precipitation rate was usually 0.1–0.2 mm/min, only in October 2016 was the intensity of rainfall higher than 0.2 mm/min per 8 min, and in October 2017 per 21 min. The torrential rainfall, which was more varied, will be described in detail.

e. Flow rates and pollutant loads transported with waters

Based on the results of numerous measurements of the amount of precipitation, the physical parameters of the watercourse bed, the flow rate, water level, and other parameters, a HEC-HMS hydraulic model of the stream flow was created^28,29^. It was calibrated using the data from the measurements of the amount of precipitation in the catchment area and the water ordinate in the retention tanks (data obtained from the local water utility Gdańskie Wody). The values of the flow rate in the watercourse in a precipitation-free time result from a series of measurements conducted on the stream. The input data for the model is the amount of rainfall. The result of the load discharged by the Oliwski Stream to the Gdańsk Bay of the Baltic Sea was calculated based on the formula (3) in point 6 (outflow to sea). Additionally, formula (4) presents the method of calculating the annual load of the analysed parameters, depending on the flow rate, pollutant concentration and the number of days with and without rainfall.

3 $${L}_{x}=\sum {Q}_{i}\cdot \overline{{c }_{x}}$$

where ${L}_{x}$—pollution load „x” [mg/d], ${Q}_{i}$—rainfall intensity in i-th hour ($i\in \left(1;24\right)$) [L/h], data obtained from the hydraulic model, $\overline{{c }_{x}}$—average concentration of pollutant "x"” [mg/L],

4 $${LA}_{x}=\sum {Q}_{i}\cdot \overline{{c }_{xd}}\cdot {n}_{d}+\sum {Q}_{i}\cdot \overline{{c }_{xr}}\cdot {n}_{r}$$

${LA}_{x}$- annual pollution load „x” [mg/d], ${Q}_{i}$—rainfall intensity in i-th hour ($i\in \left(1;24\right)$) [L/h], data obtained from the hydraulic model, $\overline{{c }_{xd}}$—average concentration of pollutant "x" in dry day [mg/L], $\overline{{c }_{xr}}$—average concentration of pollutant "x" in rainy day [mg/L], ${n}_{d}$—number of dry days (without precipitation), in Gdańsk ${n}_{d}=203$, ${n}_{r}$—number of rainy days, in Gdańsk ${n}_{r}=162$.

The results of the measurement of the flow rate in the Oliwski Stream, necessary for the calculation of pollutant loads, were calculated in the previously described HEC-HMS hydraulic model.

f. Statistical analysis and calculation models

Statistical analyzes were performed in the Statistica 13 program. Distribution normality tests were performed using the χ^2^ test. The tests verifying statistical significance was the Mann–Whitney U test.

In addition, the "Iso-Source" mixing model was used in the analysis^30–32^.

## Results and discussion

The results will be presented first with regard to the sediment analysis and then the water analysis. The analyses of the quality of the sediments were aimed at checking the sources of contamination. The surface runoff was a likely source of contamination, and its quality was therefore analysed further. Water quality analyses were aimed at checking whether the pollutant inflow phenomenon changes in different weather conditions and what is the scale of these changes.

1. The origin of carbon and nitrogen compounds in bottom sediments

**Figure 3:**
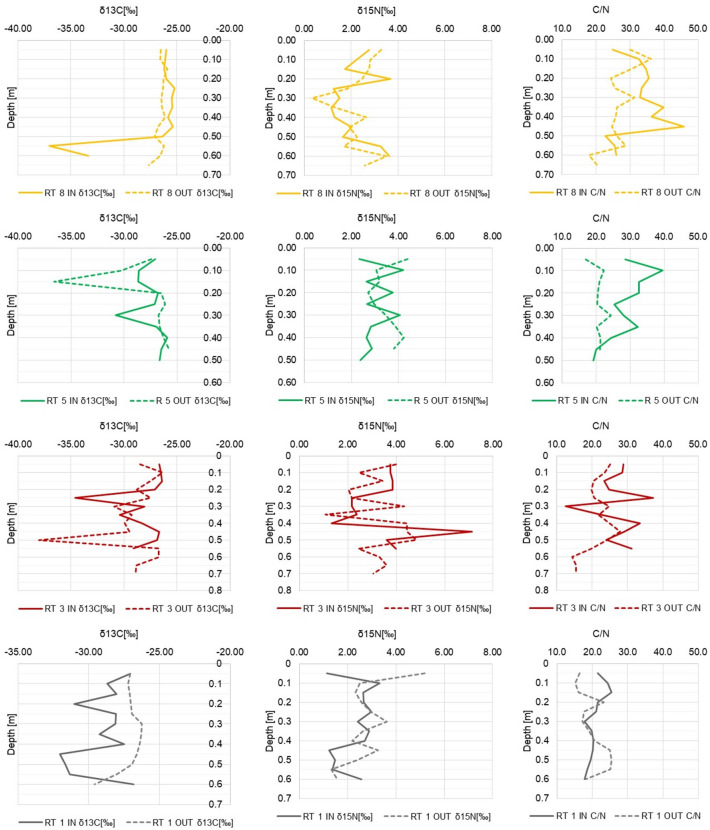
δ^13^C, δ^15^N and C/N measurement results in relation to the depth in RT8, RT5, RT3 and RT1 tanks.

The analyses of stable isotopes of carbon and nitrogen^33^ can answer the question whether organic matter is delivered to bottom sediments with stormwater or if it originates from processes taking place in the retention tank. Our analyses covered the organic carbon and total nitrogen content, as well as the carbon to nitrogen ratio^34^. Results of the isotope analyses are partly presented on Fig. 3, while detailed results are included in Appendix 1.

The ratio of carbon to nitrogen concentration can indicates the source of organic matter origin as well as providing information on processes on-going in the layer of sediments. If the ratio C/N > 12, it indicates the terrestrial origin of organic matter, whereas a ratio C/N < 8 refers to an autochthonous (planktonic) origin^35^. Zhang et al. reported that the ratio C/N = 15 is the boundary between indigenous and allochthonous origin^36^. The research conducted in China focused on answering the question of how the development of the catchment area affects the C/N ratio. It was shown that for the forest the ratio was 10.84 ± 0.11, for the meadows 10.35 ± 0.13, and for arable land 10.00 ± 0.30^37^. In our study, the median ratio C/N was 24.30, while the min–max range was 12.54–45.81. This indicates that in all RTs the organic matter was of allochthonous origin. In the surface layers of RTs 1, 3, and 5, a lower ratio occurred at the outflow than in the inlet. Statistically significant results were confirmed only for the RT5 tank., which shows that in this reservoir organic matter is probably being transformed in the surface layer of the sediments.

**Figure 4:**
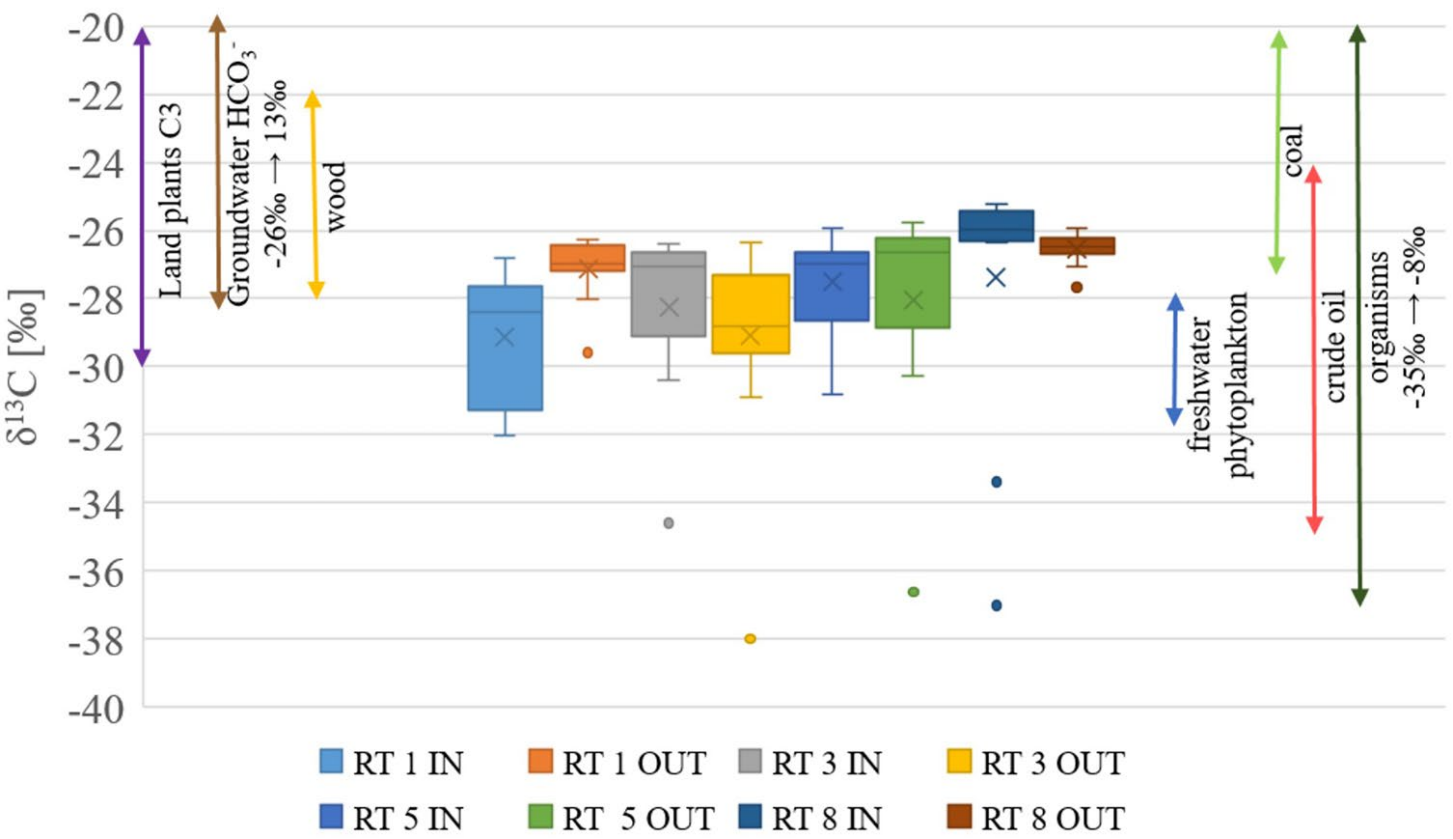
Results of δ^13^C measurements in RT bottom sediments, along with the literature data on the source of organic carbon in the sediments^46–48^.

**Figure 5:**
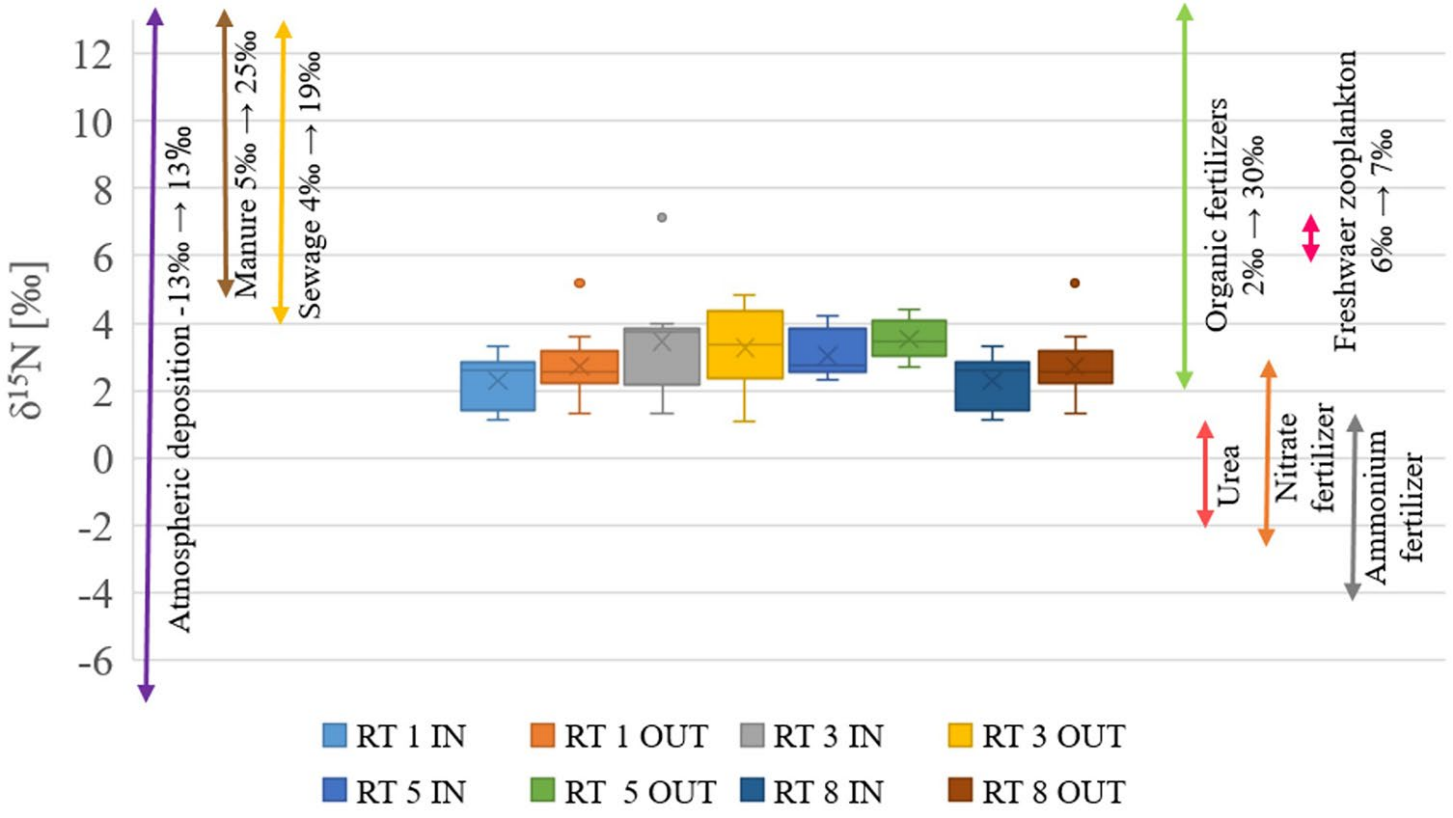
Results of δ^15^N measurements in RT bottom sediments, along with the literature data on the sources of nitrogen in the sediments^38,50–52^.

More detailed information on the specific sources of organic matter in bottom sediments can be derived from the analysis of the stable isotopes δ^13^C and δ^15^N. The results of these analyses, along with the contents of total nitrogen and organic carbon, are presented in Tables 4 and 5. The obtained results of isotope measurements in sediment samples were compared with the results of previously measured sources available in the literature^34,38,39^. Figures 4 and 5 show the ranges of results for all 8 sampling points, along with data on sources of nitrogen such as fertilizers, atmospheric deposition and sewage, as well as coal, wood and plants for carbon.

According to previous research studies, the values of δ^13^C in the range − 28‰ to − 26‰ indicate land origin (allochthonous) while the values in the range − 22‰ to − 19‰ point out aquatic (autochthonous) source^40,41^. Referring to these reports, it can be assumed that most of the organic matter in bottom sediments from RTs on Oliwski stream was of land origin, while the remainder originated from freshwater phytoplankton and type C3 terrestrial plants (that can bind CO_2_ directly from the atmosphere and photosynthesis following the Calvin-Benson path)^34,37^.

**Figure 6:**
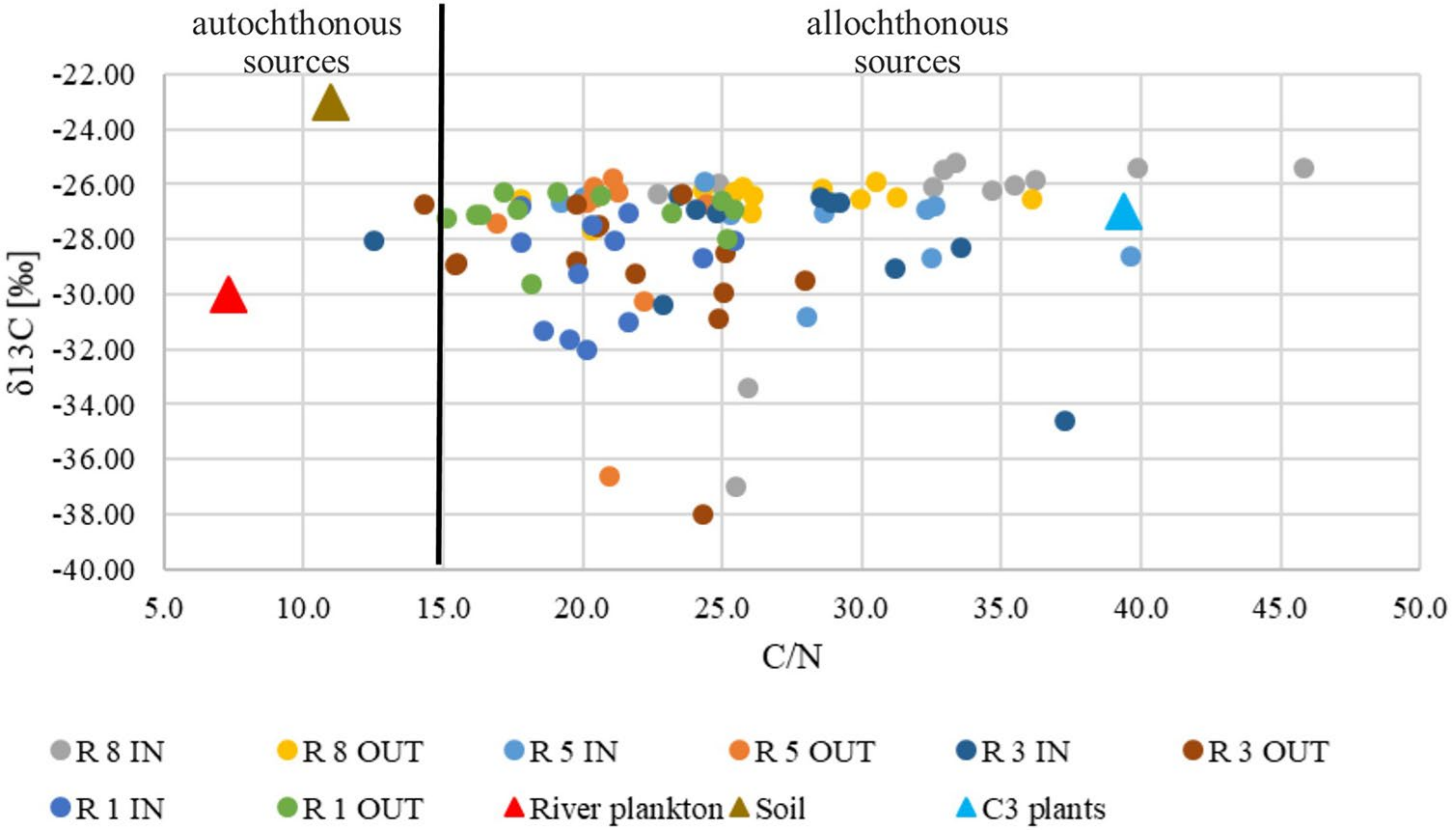
Scatterplots of δ^13^C versus C/N for potential sources of organic carbon, R-retention tank, IN-inflow, OUT-outflow^36^.

A diagram presenting the relationship between C/N and δ^13^C was made to confirm the origin of organic matter on the basis of autochthonous and allochthonous sources (Fig. 6). The values reported in the previous research studies were also marked on the diagram. Values of δ^13^C and C/N for freshwater plankton are respectively − 30.0 ± 2‰ and 7.3^42,43^, while for soils, respectively – 23.29 ± 1.39 ‰ and 10.92 ± 1.82^43^. For C3 plants (in the analyzed region these could be, for example, willows and pines) δ^13^C was − 27.12 ± 1.75‰ and C/N ratio was 39.37 ± 21.71^44,45^, while for C4 plants (including grasses, sedges, sugarcane) δ^13^C was − 13.00 ± 0.50‰, and C/N ratio was 25 ± 10^36^. The figure shows results for each tank, according to which almost all results indicate an allochthonous source of organic matter in sediments.

The results of the δ^13^C measurements are in line with the previous research reports in terms of lower values for terrestrial water reservoirs compared with the results from marine samples^33,49^. The isotope analysis indicates mixed sources of organic carbon in the analysed sediments, including crude oil , C3 land plants, groundwater, coal,wood and freshwater phytoplankton^47^. Moreover, the results of our study also fall within the range, indicating that organisms could be the carbon source^46^. Sources such as atmospheric deposition, marine plankton or marine carbonate were excluded.

An even more detailed analysis can be carried out regarding nitrogen isotope contents. As shown in Fig. 5, the range of δ^15^N contents for all tanks was in the range of 0.35–7.13. The lower values relate rather to the RT8, while the higher to RT3. This observation confirms the dependences noted by Voss et al.^53^ that higher delta values refer more to urban areas than to areas covered with forest (such as the RT8 catchment). The nitrogen sources seem to be mixed, mainly consisting of fertilizers flowing into the tanks. The amount of nitrogen in the sediments of the reservoirs was rather a mixture of organic (approx. 83%) and inorganic (approx. 17%) fertilizers. It is difficult to identify with high accuracy which types of fertilizers were in a higher dose, but considering the values, they were more likely to be nitrate fertilizers (on average 68%)^34,49^. Only in RT5 did the results clearly show 100% organic fertilizers. Discharge of sewage or the use of manure as the source of nitrogen in the sediments can be excluded.

An attempt has been made to compare the results to a few studies that were carried out in Poland and other countries. Unfortunately, data from studies of urban catchment areas are still missing, so the comparison applies to forest and agricultural catchment areas. Organic carbon concentrations—measured in the Oliwski stream—RTs do not differ much from those measured in other parts of Poland and in other countries. In six dam reservoirs in south-eastern Poland (Rzeszów, Maziarnia, Besko, Nielisz, Chańcza, and Klimkówka), the percentage of organic carbon in total organic matter varied within the range 0.08–5.90%^40^, while in Solina and Myczkowce reservoirs it was between 1.94–2.92%, and 3.95–4.08%, respectively^54^. In France, in the Kervida–Naizin catchment area, the obtained results also did not exceed 5.80% in the collected cores^55^.

Finally, correlations between δ^13^C and δ^15^N for all reservoirs were also analysed (Table 6). A significant correlation with *p* < 0.05 occurred in two reservoirs: RT8 and RT3. After splitting the results into the inflow and outflow, it was concluded, that in the RT8 tank, the correlation occurred only for the inflow (value 0.63), while in the RT3 tank for the outflow (value 0.59) (Table 2). The existing correlation indicate a dominance of a common source of organic matter in the sediments.

**Table 2:** Correlation between δ^15^N and δ^13^C in four retention tanks in Gdańsk.

Correlation	RT 1 IN δ^15^N [‰]	RT 1 OUT δ^15^N [‰]	RT 3 IN δ^15^N [‰]	RT 3 OUT δ^15^N [‰]	RT 5 IN δ^15^N [‰]	RT 5 OUT δ^15^N [‰]	RT 8 IN δ^15^N [‰]	RT 8 OUT δ^15^N [‰]
RT 1 IN δ^13^C [‰]	0.17	–	–	–	–	–	–	–
RT 1 OUT δ^13^C [‰]	–	0.56	–	–	–	–	–	–
RT 3 IN δ^13^C [‰]	–	–	0.48	–	–	–	–	–
RT 3 OUT δ^13^C [‰]	–	–	–	− 0.59	–	–	–	–
RT 5 IN δ^13^C [‰]	–	–	–	–	− 0.26	–	–	–
RT 5 OUT δ^13^C [‰]	–	–	–	–	–	− 0.02	–	–
RT 8 IN δ^13^C [‰]	–	–	–	–	–	–	− 0.63	–
RT 8 OUT δ^13^C [‰]	–	–	–	–	–	–	−	− 0.12

**Figure 7:**
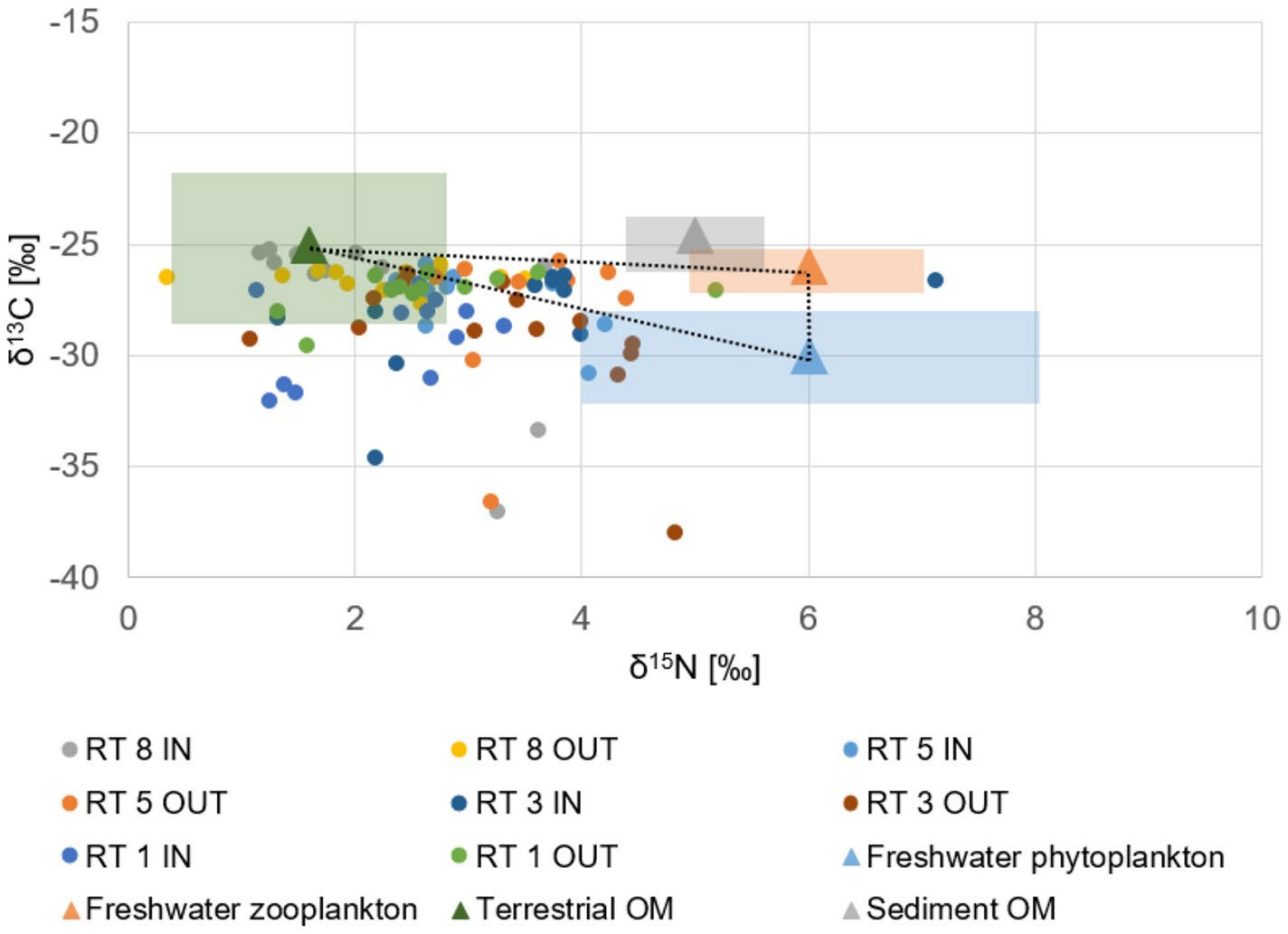
Mixing plot for δ^15^N and δ^13^C from three potential sources for all samples. Triangles indicate values for 4 sources, while rectangles indicate typical ranges of isotope values^2,43,56,57^.

**Figure 8:**
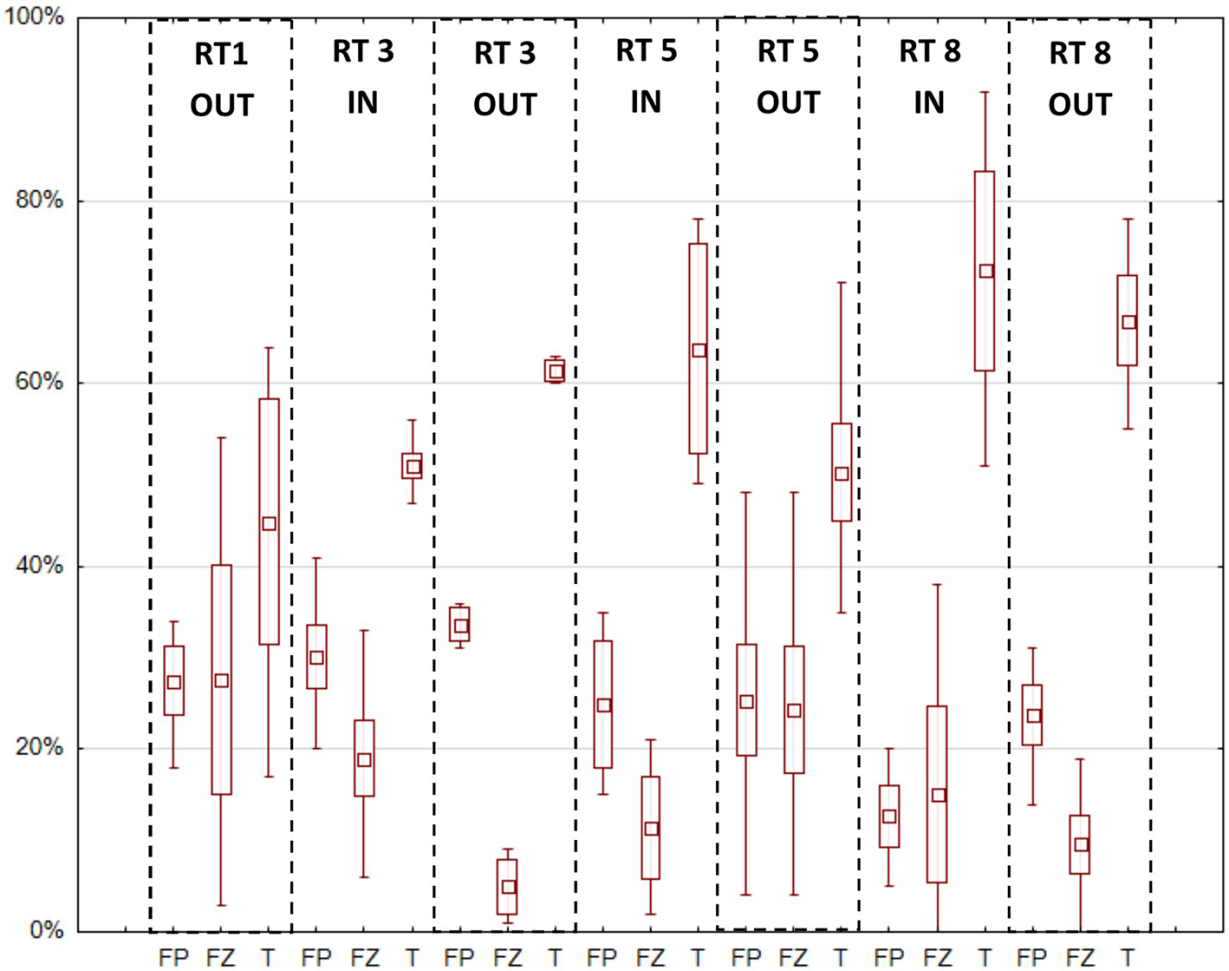
Share of mixed sources in successive retention reservoirs (RT) at the inflow (IN) and outflow (OUT), respectively. Three sources were distinguished: FP—Freshwater phytoplankton, ZP—Freshwater zooplankton, T—Terrestrial OM. Analysis performed for 24 points estimated using a multiple source mixing model (“Iso-Source”). The midpoint is the average and the whiskers indicate the min–max range.

A mixing graph for two data was also made for four sources of organic matter and nitrogen in the sediments: freshwater zooplankton, freshwater phytoplankton, terrestrial OM and sediment OM (Fig. 7). According to the graph, sedimentation was not the source of the carbon and nitrogen. Some of the samples probably came from mixed sources, so a triangle was made between the three sources, and there were 24 samples in it (3 in RT1, 6 in RT3, 8 in RT5 and 7 in RT8). Figure 8 presents the share of individual sources (FP, FZ, T) in the sediments of four reservoirs (RT). In the tanks RT3, RT5 and RT8, the highest content of terrestrial sources was confirmed (in the ranges of 47–63%, 35–78% and 51–92%, respectively). The highest share of Terrestrial OM was recorded in sediment samples from RT 5 IN and RT 8 IN, up to 78% and 92%, respectively. However, the highest share of FZ was recorded in the RT1 OUT and RT5 OUT samples (54% and 48%). Also in the RT5 OUT (as well as RT3 IN) samples, the FP content was high, up to 48% and 41% respectively.

In the above considerations, it has been proved that the pollutants collected in the sediments partly come from the surface runoff from the catchment area. Therefore, in the following part, the weather in which the volume of pollutants were the highest was checked. The results from the periods of floods were particularly interesting, because they are a relatively rare phenomena, and yet in the analyzed period they occurred twice, and their course was completely different. The second part of the results concerns water quality analyses, uses hydrological measurements and is aimed at assessing various weather phenomena in relation to the water pollution of the watercourse.

2. Rainfall characteristics

**Figure 9:**
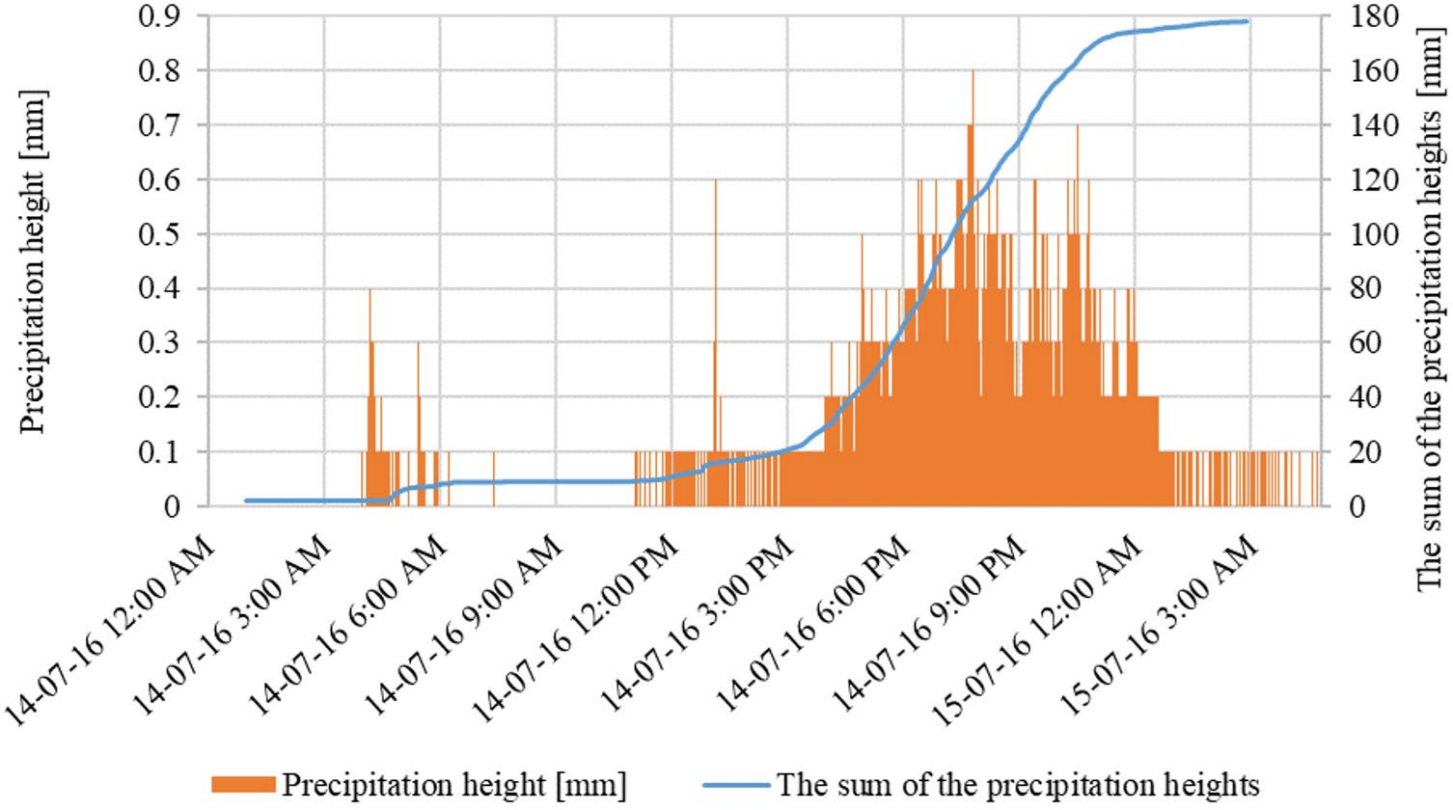
Total precipitation curve, measured in Gdańsk Oliwa on July 14–15th, 2016.

**Figure 10:**
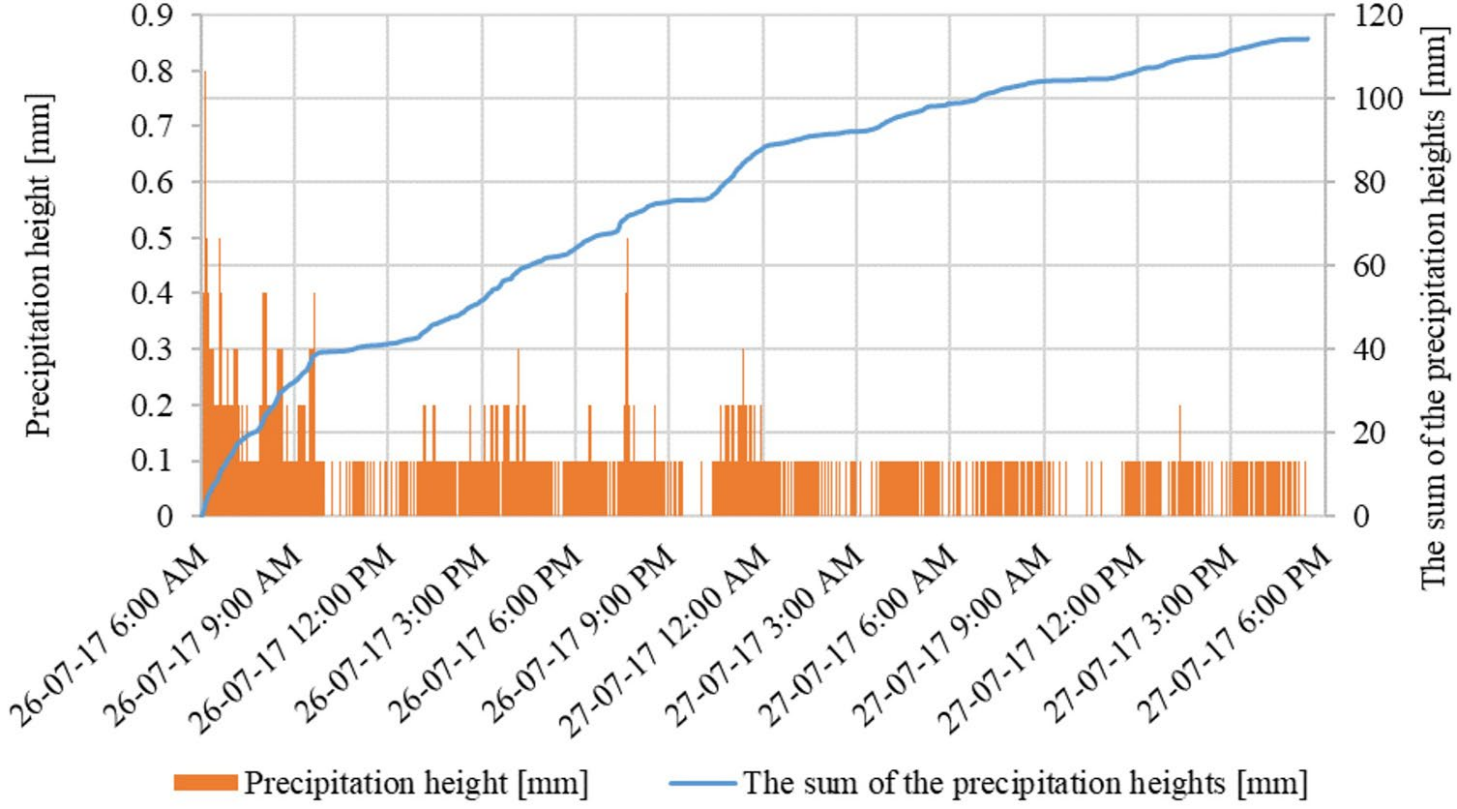
Total precipitation curve, measured in Gdańsk Oliwa on July 26–27th, 2017.

The rainfall in July 2016, started on Thursday the 14th at 1:00 AM, but before 05:00 AM crossed a depth of 5 mm, while its intensity began to increase at approximately 12:00 AM. The maximum intensity was recorded at 0.8 mm within 1 min, 16.1 mm in 30 min, and 27.3 mm in an hour, between 6–7 PM (when the amount was 89.1 mm). Precipitation stopped before 3:00 AM on July 15th. The total precipitation depth was recorded at 178.1 mm (Fig. 9). In 2017, the precipitation scenario was different. The rainfall began around 6:00 AM on July 26th with an average intensity of 7.5 mm in 30 min. The maximum intensity of precipitation was also 0.8 mm within 1 min and 14.9 mm in 30 min, but this maximum intensity was much shorter than in July 2016. The precipitation ended on July 27th at about 5:30 PM. The total precipitation depth was 114.3 mm (Fig. 10).

The rainfall that occurred in Gdańsk on July 14–15, 2016, and July 26–27, 2017, caused numerous floods, which were extremely severe in the catchment area of Oliwski Stream. The characteristics of both rainfall events have been presented in Table 3. The rainfall in July 2016 was classified as heavy, although periodically it showed a torrential character^58^. The amount of precipitation which occurred on 14 and 15th of July 2016 was close to the two-month precipitation norm for the analysed area. As a result of this rainfall, the city area suffered significant damage, including residential and educational buildings, streets, and sidewalks. The tunnels, tram tracks, and trams were flooded. Two retention reservoirs were also damaged (including one on the Oliwski Stream). Losses after precipitation in 2016 were estimated at 10.5 million PLN (2.65 M USD). The flood in 2017 did not cause such large losses due to the lower intensity of rainfall and the experience from a year ago (a larger capacity in retention reservoirs, sandbags for buildings, construction of flood dams). The only consequence of the torrential rains was the flooding of streets and sidewalks.

**Table 3:** Torential rainfall characteristicsWater quality after rainfall events.

Parameter	July 14/15, 2016	July 26/27, 2017
Precipitation height [mm]	178.1	114.3
The sum of precipitation in the calendar year [mm]	766.5	893.9
Share of precipitation amount in the annual precipitation [%]	23.2	12.8
The duration of the precipitation [h]	14	34

Since the isotope analyses undoubtedly indicated the allochtonous sources of nitrogen and organic carbon in sediments, stormwater runoff seemed to be the most likely source of C and N compounds. Therefore, we analysed the water quality changes in dry and wet weather, with special regard to two torrential rainfall events that occurred during the study period. Stormwater flowing in the stream bed (delivered with runoff) transports dissolved and suspended pollutants. The largest inflow to the tanks occurs during or after the rainfall. The amount of rainfall is also important, and more intense rainfall is likely to be the source of the higher pollutant load (both because of the higher concentration, but above all because of the higher flow rate). Analysis of the water quality in Oliwski stream is presented in Table 4. From presented results, it can be concluded that after torrential rains most of the specific concentrations were significantly higher (up to 8 times) than after "typical" precipitation. The influence of the amount of precipitation was far more noticeable in relation to nitrogen compounds, especially in July 2016, when the amount and intensity of the downpour was higher, leading to an observation that leaching of nitrogen compounds is greater during more intense rainfall. Statistical analyzes confirmed that, apart from N-NH_4_ in 2016 and P-PO_4_ in 2017, the concentrations of the tested compounds after torrential rainfall increased (*p* < 0.05).

**Table 4:** Characteristics of water quality [mg/L] near the mouth (point 6) during 3 types of weather.

Parameter	Dry weather	Wet weather	Torrential rain	
			2016	2017
N-NO_2_	0.018	0.019	0.070	0.040
N-NO_3_	0.62	0.78	1.37	0.96
N-NH_4_	0.09	0.12	0.14	0.13
P-PO_4_	0.05	0.1	0.18	0.04
Ptot	0.09	0.07	0.18	0.04
COD	8.6	11.4	22.5	26.8
TSS	55.2	53.7	435.7	69.1

**Figure 11:**
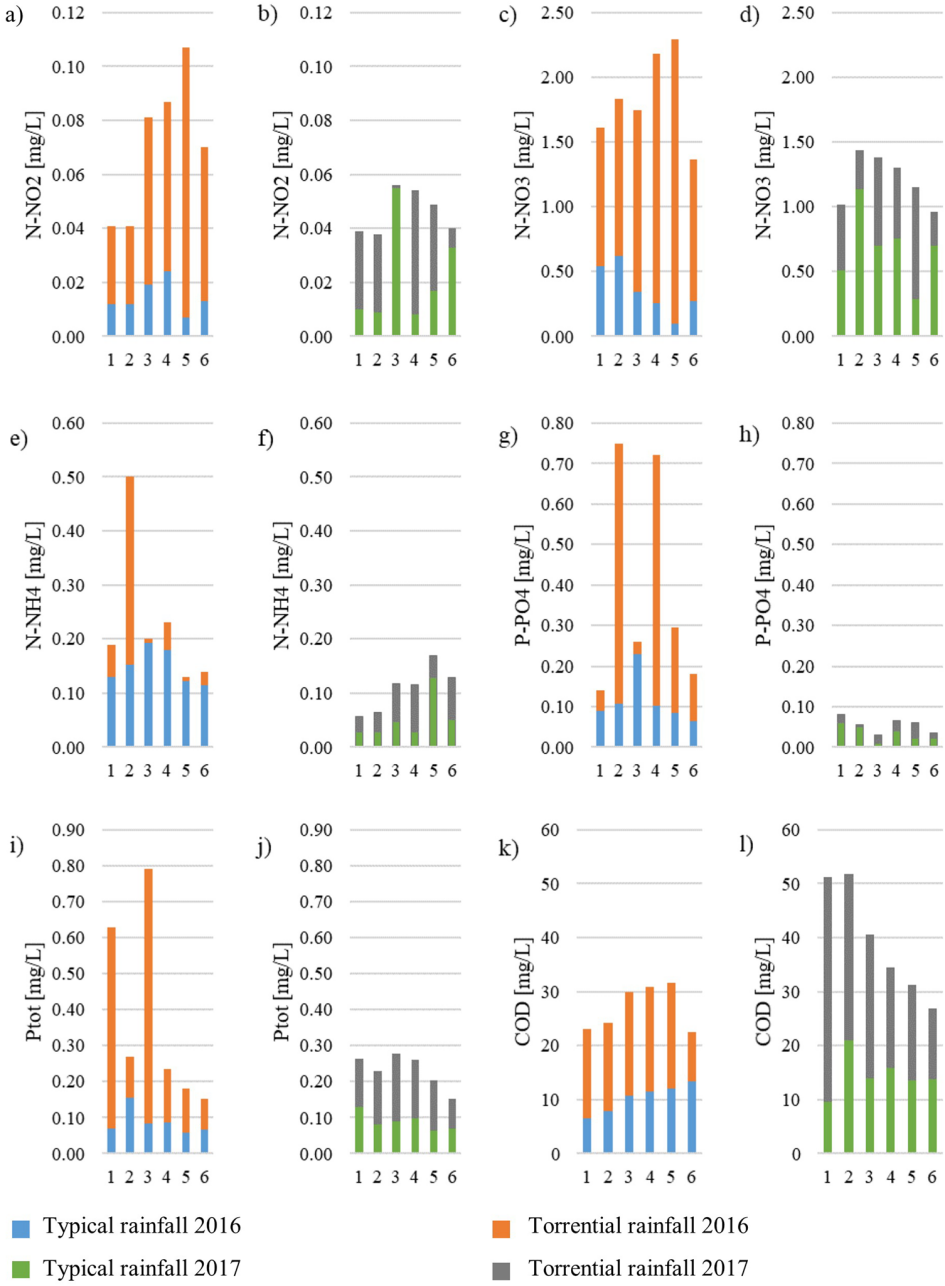
Concentrations of nitrogen forms (N–NO_2_, N–NO_3_, N–NH_4_), phosphorus (P–PO_4_, Ptot) and COD in Oliwski stream during urban floods in July 2016 and July 2017. The blue/green bars represent the results after typical rainfall (June 2016 and 2017), and the entire bars (the sum of blue and orange or green and grey) refer to the results during torrential rainfall in June 2016 and June 2017.

**Figure 12:**
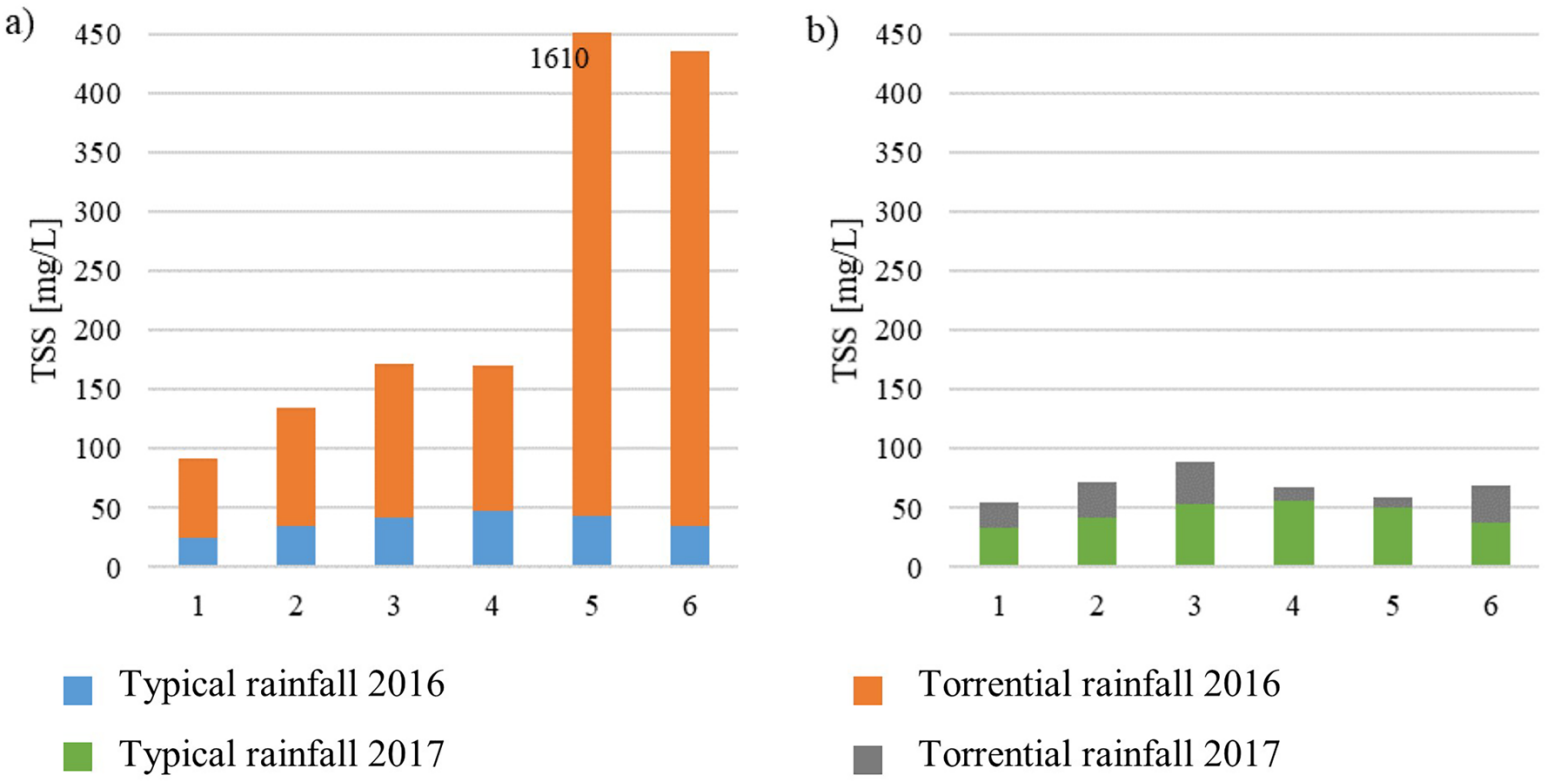
Concentrations of TSS in Oliwski stream during urban floods in July 2016 and July 2017. The blue/green bar represents the result after typical rainfall (June 2016 and 2017,) and the entire bar (the sum of blue and orange or green and grey) is the July result.

In the charts below (Figs. 11 and 12), changes in water quality after torrential rainfall in 2016 and 2017 are presented, with regard to the “typical” rainfall events that occurred in a similar time period (June 2016, June 2017). Precipitation after which samples were taken in June 2016 was 30.0 mm, while the typical rainfall in June 2017 was 23.5 mm, which represents 17% and 21% of the sum of the torrential rainfalls in 2016 and 2017, respectively. Torrential rainfalls caused an increase in the concentrations of all measured parameters, from 3 times increase of P-PO_4_ in July 2017 to an over 124 times increase of TSS in July 2016 in relation to wet weather. The highest increase, on average, was 5 times for N–NO_3_ and almost 5 times for N-NO_2_, and the smallest (on average 2 times) for N–NH_4_. A significant increase in the concentration of N–NO_3_ and TSS after torrential rainfall in 2016 was observed in point 5, which, however, was the result of damage to the tank located upstream of the sampling point. As a result, part of the sediments was removed in the form of a suspension along with the flowing water. Figures 11 and 12 show that after the rainfall in 2016, the pollutant concentrations were higher than in 2017. The analysis was statistically confirmed for N–NO_3_, N–NH_4_, P–PO_4_, COD and TSS (*p* < 0.05). This is probably linked to a greater sum of precipitation that caused a more rapid runoff and, subsequently, damage to the retention tank, which resulted in an ucontrolled spill.

The results of the Spearman correlation for the torrential rainfalls in July 2016 and 2017 showed that TSS concentration was correlated with the concentration of N-NO_2_ and N-NO_3_, with correlation coefficient values equal to 0.75 and 0.61, respectively (Table 5). Considering only points 1–4 (upstream of the retention tank failure), the values of the coefficients for nitrogen compounds were higher. In earlier studies, a correlation between P-PO_4_ and TSS concentration was demonstrated (coefficient value 0.75)^59–61^. It was observed that during typical rainfall (as in June 2016 and 2017) the concentrations of contaminants decreased with the flow direction, after flowing through successive retention tanks, which may indicate a greater quantity of removal via sedimentation in tanks. This is also confirmed by the analysis of the correlation coefficient, according to which the concentration of TSS was related to the concentration of not only N-NO_2_ and N-NO_3_ but also Ptot and COD, the correlation coefficients in the case of wet weather were higher (values 0.84, 0.78, 0.96, 0.73, respectively) (Table 5).

**Table 5:** Correlations between the concentrations of TSS and the concentrations of nitrogen compounds, phosphorus, and organic matter in water samples.

Parameter	Torrential rainfall (points 1–6)	Torrential rainfall (points 1–4)	Typical rainfall in June 2016 (points 1–6)
	TSS	TSS	TSS
N–NO_2_	0.75	0.79	0.84
N–NO_3_	0.61	0.90	0.78
N–NH_4_	− 0.07	0.60	0.38
P–PO_4_	0.15	0.75	0.52
Ptot	− 0.21	0.40	0.96
COD	− 0.16	− 0.61	0.73

3. The load of pollutants

Determination of the loas of pollutants flowing into the Baltic Sea by the waters of the Oliwski stream was made for the sampling point no. 6, located directly at the mouth of the watercourse into the Bay of Gdańsk. The pollutant loads after normal and torrential rainfall and in the period without rainfall were calculated in accordance with formula (2). The flow rate at the closing point of the watercourse was dependent on the weather. During dry weather, it was equal to about 0.22 m^3^/s, in wet weather (depend on the momentary intensity of precipitation and runoff) it was in the range of 0.23–6.17 m^3^/s, on average 1.95 m^3^/s, and the median was 1.55 m^3^/s. During the torrential rainfall in July 2016, the flow intensity in the stream varied from 0.52 to 112.91 m^3^/s, with an average of 30.12 m^3^/s and a median of 9.25 m^3^/s. Whereas during the torrential rainfall in July 2017 the volatility was less, the flow intensity in the stream varied from 0.74 to 22.75 m^3^/s (average of 13.19 m^3^/s, median of 13.61 m^3^/s).

**Figure 13:**
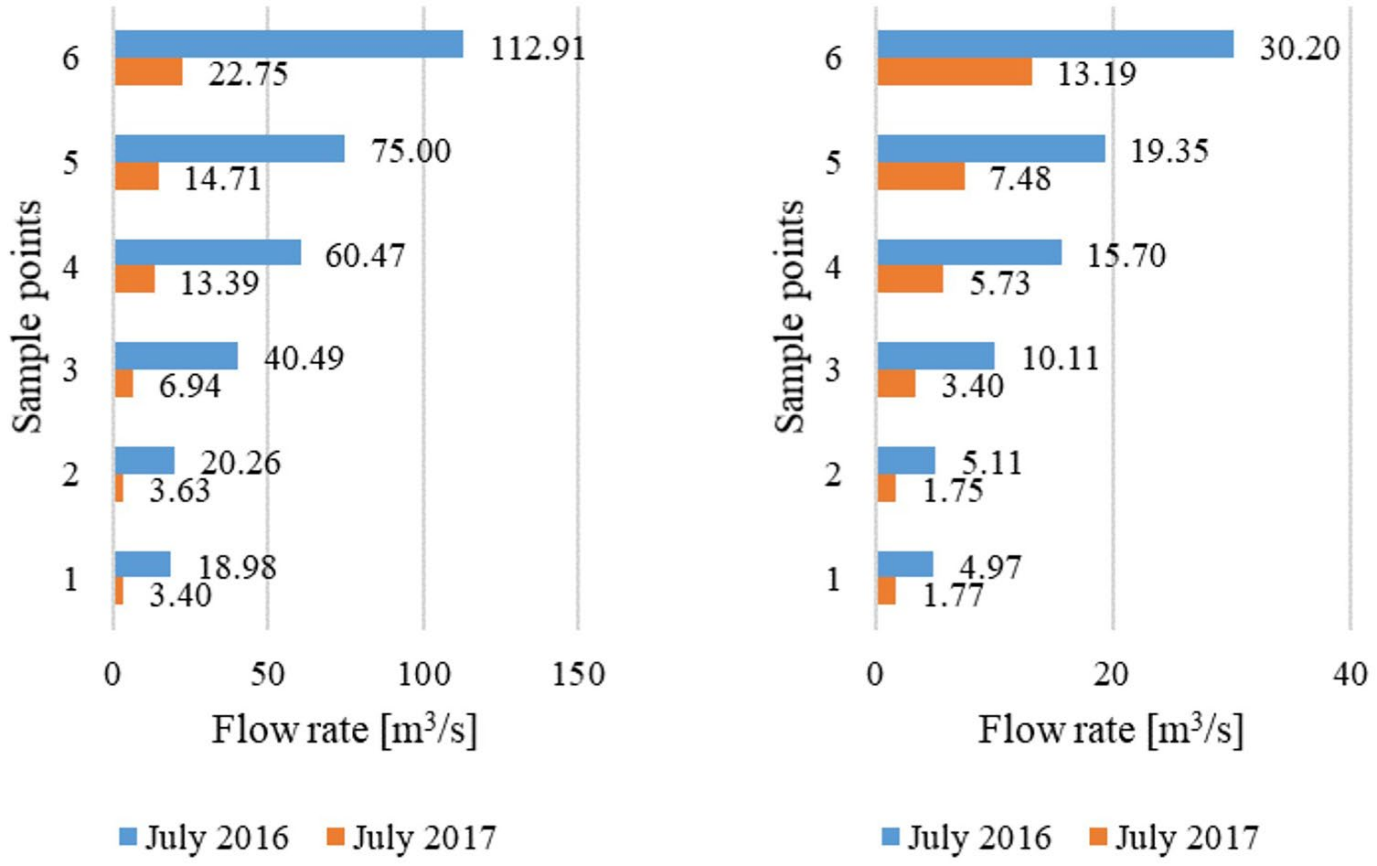
The flow rate at points 1–6 of the Oliwa stream after torrentail rainfall in July 2016 and July 2017. (**a**) Maximum flow rate, (**b**) average flow rate.

Additionally, a comparison of the flow rate in the watercourse after two torrential rainfall events (Fig. 13) indicates that at all sampling points maximum flow values were more than 5 times higher after the torrential precipitation in 2016, compared within the rainfall episode in 2017. The mean values were 2.3–3.0 times higher during the torrential rainfall in 2016 compared with the rainfall in the following year. These differences are mainly due to higher precipitation intensity and longer rainfall duration in 2016 than in 2017.

**Figure 14:**
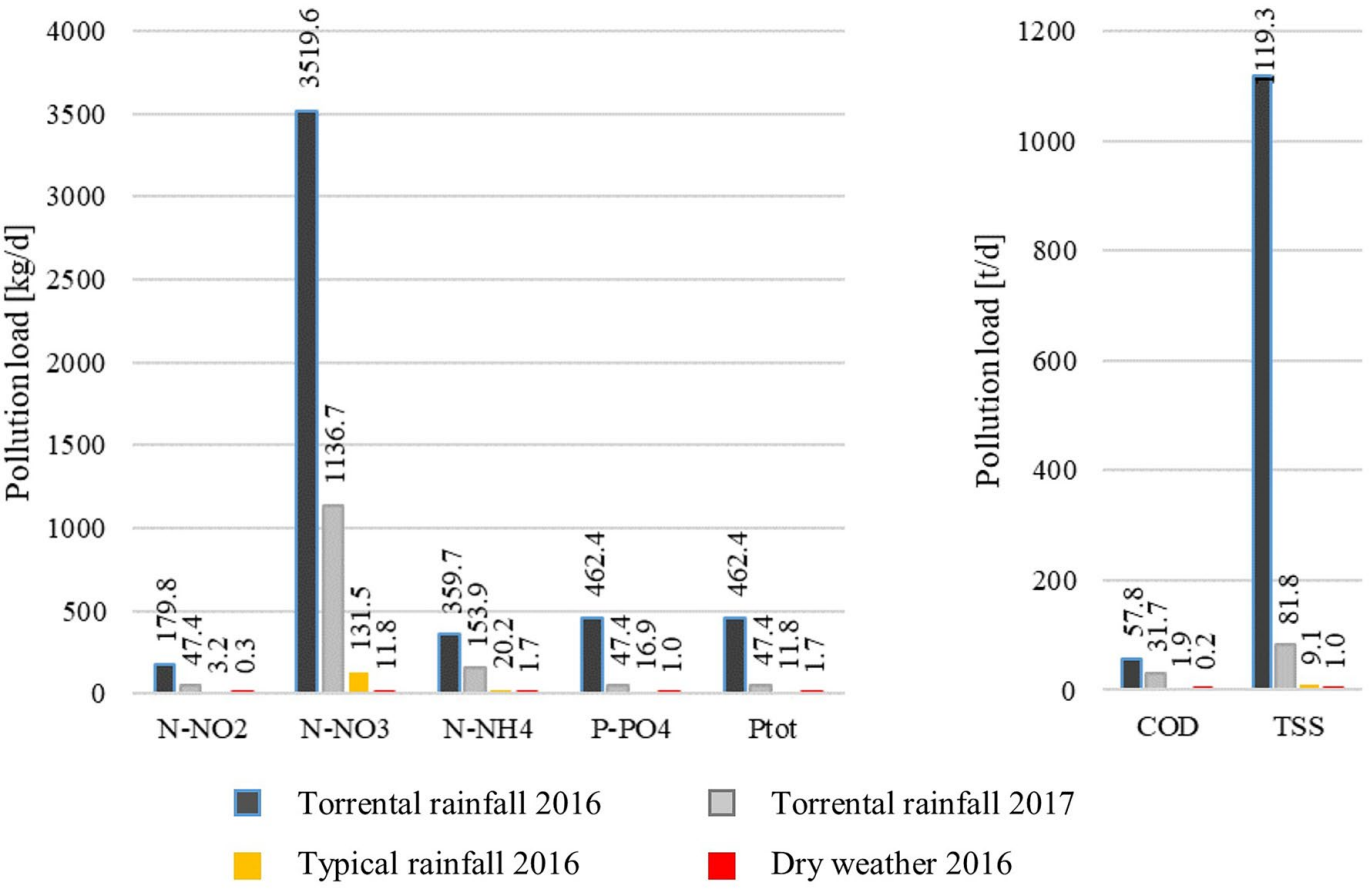
Loads of N–NO_2_, N–NO_3_, N–NH_4_, P–PO_4_, Ptot, COD, and TSS discharged into the Baltic Sea during dry weather, typical rainfall, and torrential rainfall.

The volume of pollutants discharged after torrential rainfall in comparison with the volume in dry weather and wet weather arehown in Fig. 14. Additionally, Table 6 presents the results of calculations of the annual loads of pollutants flowing into the Baltic Sea. The yearly average number of rainy days in Gdańsk is 162, with 203 rainless days. These numbers, together with the daily pollutant loads calculated earlier, make it possible to estimate the approximate annual load discharged from the Oliwski stream (Table 6). The volume of pollutants discharged after rainfall in July 2016 accounted for 67% of the annual load of TSS, 31% N–NO_2_, and 10–20% of other analyzed parameters. After the rainfall in July 2017, the discharged load was considerably lower; even so, it accounted for 2–9% of the yearly discharge of analyzed pollutant loads. As a result of the flood which occurred in July 2016, an extra volume of over 3.5 tonnes of N–NO_3_, over 57 tonnes of organic matter (expressed as COD), and over 1100 tonnes of TSS were discharged into the Baltic Sea. This sole flooding event was responsible for 2.4% of the annual load of N–NO_3_ allowed from Polish territory by HELCOM’S Baltic Sea Action Plan (BSAP)^62^. Such high loads must be significant for the environment. The flooding does not only cause material losses in urbanized areas, a matter that can be solved with appropriate financial measures, but also, and even more importantly, losses to the environment, which can be irreversible, especially in the context of Baltic eutrophication and hypoxia, leading to the formation of a dead zone.

**Table 6:** Comparison of annual loads of pollutants discharged by the Oliwski stream and the loads discharged due to pluvial floods caused by one day of torrential rainfall in 2016 and 2017.

Parameter		Annual load	July 2016		July 2017	
			A one-day load	Annual load share (%)	A one-day load	Annual load share (%)
N–NO_2_	[kg/year]	588	179.8	31	47.4	8
N–NO_3_		23,695	3519.6	15	1136.7	5
N–NH_4_		3625	359.7	10	153.9	4
P–PO_4_		2924	462.4	16	47.4	2
Ptot		2259	462.4	20	47.4	2
COD	[t/year]	345	57.8	17	31.7	9
TSS		1680	1119.3	67	81.8	5

**Figure 15:**
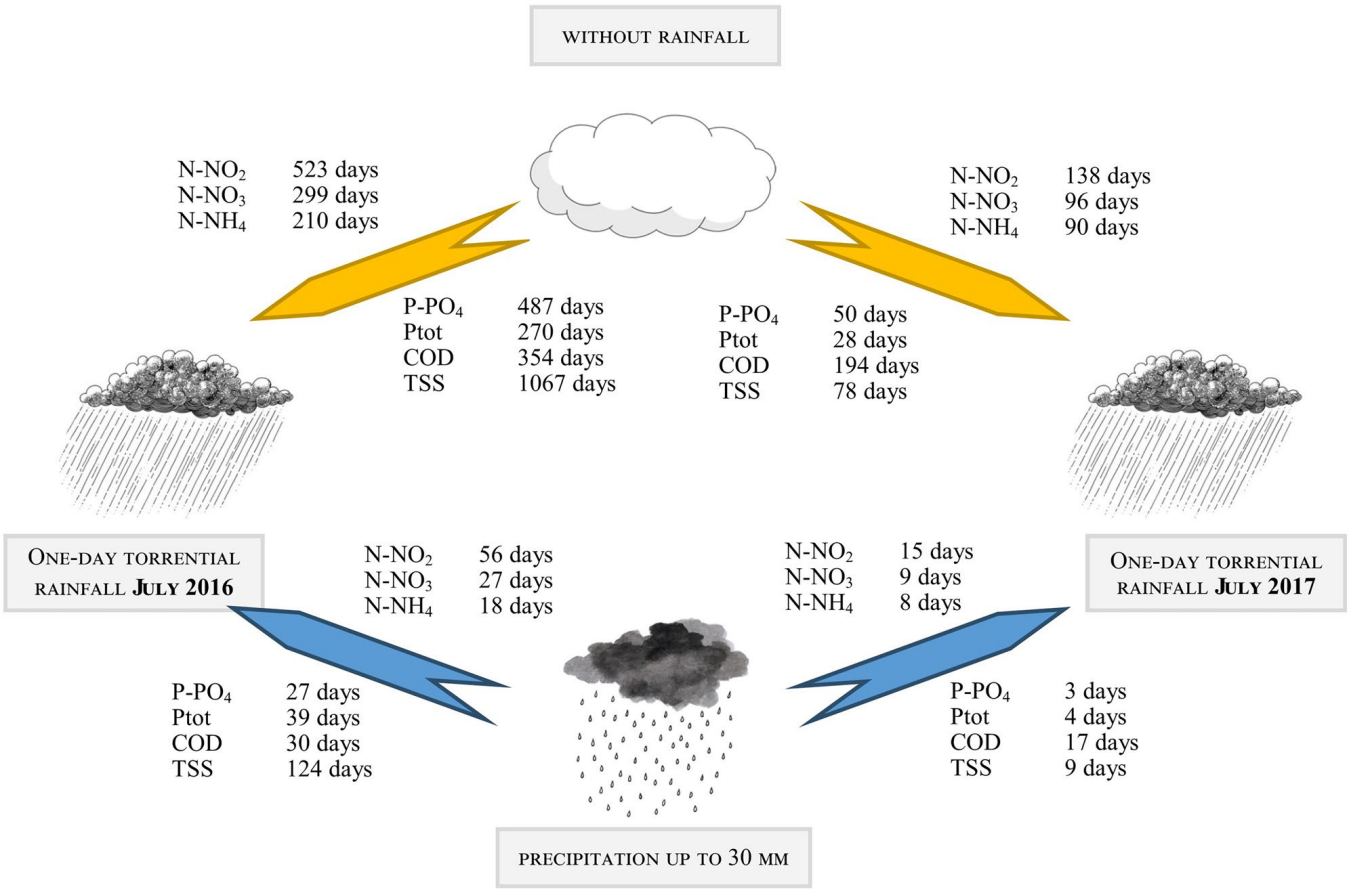
The number of days without precipitation and with precipitation up to 30 mm, which result in the discharge of the same load of pollutants that outflowed after the torrential rainfalls in July 2016 and July 2017. Drawing analysis: how many days without rainfall (at the top of the drawing) or with wet weather (at the bottom of the drawing) are needed to remove the same amount of pollutants as after torrential rainfall in 2016 (left) and after torrential rainfall in 2017 (right).

Figure 15 shows the results of the calculations – the number of days, respectively, without precipitation or with wet weather, which would result in the same load as that discharged after torrential precipitation. For example, in case of N–NO_2,_ it would take 17 months of dry weather to discharge the same load as was daily emitted during a torrential rainfall event in July 2016. For P–PO_4_, and TSS, it would be 16 months, and almost 3 years, respectively. After the torrential rainfall in 2017, the time periods were lower: 4.5 months in relation to N–NO_2_, more than 6 months in relation to COD, more than 3 months in relation to N–NO_3_ and N–NH_4_. For comparison, the period with "typical" precipitation, in relation to nitrogen compounds and COD, was about 10 times shorter, in relation to P–PO_4_ and Ptot 17 and 7 times shorter, respectively. This illustrates the significance of stormwater runoff from urban areas to the quality of surface waters, and especially emphasizes the great impact of catastrophic rainfalls and urban floods, which is often downsized or neglected.

## Conclusions

Our research helps to elucidate the pathways of non-point pollutants migration as well as contributing to the assessment of complex phenomena of pollutant deposition, re-suspension, and transport in urban stormwater receivers and retention tanks, especially during pluvial floods.

Due to the dynamically increasing population and urban density, pluvial floods risk dramatic increases. Urban floods can bring severe consequences not only in terms of material losses but can also cause significant discharge of pollutants which pose a substantial risk to the environment. Therefore, it is important to understand the response of urban areas in terms of pollutants released during rainy weather.

By means of an analysis of stable isotopes of carbon and nitrogen, we identified the allochthonous (terrestrial) origin of pollutants deposited in the sediments, which indicated that the pollutants were brought to the retention tanks with stormwater runoff. The allochthonous source was confirmed not only by direct analyses, but also by analyzes of samples indicating a mixed origin of contaminants using a multi-source mixing model. Organic nitrogen fertilizers turned out to be the major source of nitrogen in bottom sediments. In contrast, the sources of organic carbon were mixed, including land C3 plants, wood, and oil. Furthermore verified was a minor contribution from freshwater phytoplankton.

A comparison of concentrations of N–NO_2_, N–NO_3_, N–NH_4_, P–PO_4_, Ptot, COD, and TSS after torrential and "typical" rainfalls (with a precipitation depth of up to 30 mm) revealed a significant increase in all concentrations measured after torrential rainfall events. We observed the following increases: N–NO_3_ 1.2-fold, N–NH_4_ 1.8-fold, P–PO_4_ about 2.0-fold, Ptot about 2.0-fold, COD about 2.0-fold, and TSS 8.0-fold. This applies to nitrate and nitrate nitrogen, the concentration of which increased by 3.7 times after torrential rainfall compared with wet weather. The retention tanks were less effective in reducing the inflowing volume of pollutants during torrential rainfall than during typical rainfall. This was due to the limited sedimentation under greater flow dynamics, which could also cause re-suspension of already deposited pollutants. The loads of nitrogen and phosphorus compounds discharged by Oliwski stream after one day of torrential rainfall corresponded to loads discharged during 1 year in the case of nitrogen, and 3–4 months in case of phosphorus, during dry weather. Referring to “typical” precipitation, the same load, as that during torrential rainfall,could be discharged during the periods of 18 to 56 days of typical rainfall for nitrogen compounds, phosphorus, and organic matter loads and up to 24 days for TSS. Torrential rainfall contributed to a rapid increase in the concentration of pollutants in the stream receiving stormwater runoff.

Retention tanks play a very important role in flood prevention in urban areas. They also effectively retain pollutants carried with stormwater runoff, although they are less effective during torrential rainfall and pluvial floods. Furthermore, sediments deposited in urban retention tanks can be a source of water recontamination. Future research should focus on ways to reduce runoff to watercourses, such as an analysis of the effects of buffer zones along watercourses that should reduce runoff.

## Supplementary Information

Supplementary Information.

## Data Availability

The datasets used and/or analysed during the current study available from the corresponding author on reasonable request.
