# Supplementary material for: Sources of contamination in sediments of retention tanks and the influence of precipitation type on the size of pollution load
Source: Sci Rep. 2023 Jun 1;13:8884. doi: 10.1038/s41598-023-35568-9 (PMC10235034; doi:10.1038/s41598-023-35568-9)
Supplement: Supplementary file 1 — Supplementary Information. [file 41598_2023_35568_MOESM1_ESM.docx]

| \| **Depth** \| **N_tot_[%]** \| **δ^15^N[‰]** \| **C_org_[%]** \| **δ^13^C[‰]** \| **C/N** \| \| --- \| --- \| --- \| --- \| --- \| --- \| \| Retention tank No 8 inflow (R 8 IN) \| \| \| \| \| \| \| 0.05 \| 0.03 \| 2.76 \| 0.71 \| -25.97 \| 24.9 \| \| 0.10 \| 0.02 \| 2.24 \| 0.45 \| -26.08 \| 32.6 \| \| 0.15 \| 0.04 \| 1.74 \| 1.07 \| -26.2 \| 34.6 \| \| 0.20 \| 0.2 \| 3.67 \| 5.95 \| -26.01 \| 35.4 \| \| 0.25 \| 0.28 \| 1.25 \| 7.96 \| -25.24 \| 33.4 \| \| 0.30 \| 0.27 \| 1.5 \| 7.75 \| -25.46 \| 32.9 \| \| 0.35 \| 0.15 \| 1.17 \| 5.1 \| -25.42 \| 39.9 \| \| 0.40 \| 0.16 \| 1.3 \| 5.1 \| -25.84 \| 36.2 \| \| 0.45 \| 0.04 \| 2.01 \| 1.4 \| -25.39 \| 45.8 \| \| 0.50 \| 0.01 \| 1.66 \| 0.25 \| -26.34 \| 22.7 \| \| 0.55 \| 0.02 \| 3.27 \| 0.49 \| -37.02 \| 25.5 \| \| 0.60 \| 0.15 \| 3.63 \| 3.37 \| -33.38 \| 25.9 \| \| **MIN** \| 0.01 \| 1.17 \| 0.25 \| -37.02 \| 22.7 \| \| **MAX** \| 0.28 \| 3.67 \| 7.96 \| -25.24 \| 45.8 \| \| **MED.** \| 0.09 \| 1.88 \| 2.39 \| -25.99 \| 33.2 \| \| **AVE** \| 0.11 \| 2.18 \| 3.30 \| -27.36 \| 32.5 \| \| **SD** \| 0.10 \| 0.89 \| 2.82 \| 3.60 \| 6.5 \| | \| **Depth** \| **N_tot_[%]** \| **δ^15^N[‰]** \| **C_org_[%]** \| **δ^13^C[‰]** \| **C/N** \| \| --- \| --- \| --- \| --- \| --- \| --- \| \| Retention tank No 8 outflow (R 8 OUT) \| \| \| \| \| \| \| 0.05 \| 0.28 \| 3.29 \| 7.07 \| -26.52 \| 29.9 \| \| 0.10 \| 0.14 \| 2.81 \| 4.34 \| -26.56 \| 36.1 \| \| 0.15 \| 0.14 \| 2.77 \| 3.71 \| -25.92 \| 30.5 \| \| 0.20 \| 0.22 \| 2.46 \| 4.66 \| -26.25 \| 24.3 \| \| 0.25 \| 0.24 \| 1.84 \| 5.32 \| -26.31 \| 25.4 \| \| 0.30 \| 0.22 \| 0.35 \| 5.81 \| -26.49 \| 31.2 \| \| 0.35 \| 0.24 \| 1.37 \| 5.42 \| -26.43 \| 26.1 \| \| 0.40 \| 0.26 \| 2.66 \| 5.81 \| -26.11 \| 25.8 \| \| 0.45 \| 0.28 \| 1.94 \| 5.88 \| -26.78 \| 24.7 \| \| 0.50 \| 0.32 \| 2.26 \| 7.13 \| -27.07 \| 26.1 \| \| 0.55 \| 0.34 \| 1.69 \| 8.35 \| -26.18 \| 28.6 \| \| 0.60 \| 0.21 \| 3.51 \| 3.28 \| -26.56 \| 17.8 \| \| 0.65 \| 0.17 \| 2.59 \| 3.00 \| -27.66 \| 20.3 \| \| **MIN** \| 0.14 \| 0.35 \| 3.00 \| -27.66 \| 17.8 \| \| **MAX** \| 0.34 \| 3.51 \| 8.35 \| -25.92 \| 36.1 \| \| **MED.** \| 0.24 \| 2.46 \| 5.42 \| -26.49 \| 26.1 \| \| **AVE** \| 0.24 \| 2.27 \| 5.37 \| -26.53 \| 26.7 \| \| **SD** \| 0.06 \| 0.81 \| 1.51 \| 0.43 \| 4.6 \| |
| --- | --- | --- | --- | --- | --- | --- | --- | --- | --- | --- | --- | --- | --- | --- | --- | --- | --- | --- | --- | --- | --- | --- | --- | --- | --- | --- | --- | --- | --- | --- | --- | --- | --- | --- | --- | --- | --- | --- | --- | --- | --- | --- | --- | --- | --- | --- | --- | --- | --- | --- | --- | --- | --- | --- | --- | --- | --- | --- | --- | --- | --- | --- | --- | --- | --- | --- | --- | --- | --- | --- | --- | --- | --- | --- | --- | --- | --- | --- | --- | --- | --- | --- | --- | --- | --- | --- | --- | --- | --- | --- | --- | --- | --- | --- | --- | --- | --- | --- | --- | --- | --- | --- | --- | --- | --- | --- | --- | --- | --- | --- | --- | --- | --- | --- | --- | --- | --- | --- | --- | --- | --- | --- | --- | --- | --- | --- | --- | --- | --- | --- | --- | --- | --- | --- | --- | --- | --- | --- | --- | --- | --- | --- | --- | --- | --- | --- | --- | --- | --- | --- | --- | --- | --- | --- | --- | --- | --- | --- | --- | --- | --- | --- | --- | --- | --- | --- | --- | --- | --- | --- | --- | --- | --- | --- | --- | --- | --- | --- | --- | --- | --- | --- | --- | --- | --- | --- | --- | --- | --- | --- | --- | --- | --- | --- | --- | --- | --- | --- | --- | --- | --- | --- | --- | --- | --- | --- | --- | --- | --- | --- | --- | --- | --- | --- | --- | --- | --- | --- | --- | --- | --- | --- | --- | --- | --- | --- | --- | --- | --- | --- | --- | --- | --- | --- | --- |
|  |  |
| \| **Depth** \| **N_tot_[%]** \| **δ^15^N[‰]** \| **C_org_[%]** \| **δ^13^C[‰]** \| **C/N** \| \| --- \| --- \| --- \| --- \| --- \| --- \| \| Retention tank No 5 inflow (R 5 IN) \| \| \| \| \| \| \| 0.05 \| 0.14 \| 2.33 \| 3.45 \| -27.04 \| 28.6 \| \| 0.10 \| 0.19 \| 4.21 \| 6.51 \| -28.64 \| 39.6 \| \| 0.15 \| 0.29 \| 2.63 \| 8.15 \| -28.67 \| 32.5 \| \| 0.20 \| 0.21 \| 3.76 \| 5.96 \| -26.78 \| 32.6 \| \| 0.25 \| 0.32 \| 2.65 \| 6.93 \| -27.1 \| 25.3 \| \| 0.30 \| 0.29 \| 4.07 \| 6.88 \| -30.82 \| 28.0 \| \| 0.35 \| 0.08 \| 2.82 \| 2.1 \| -26.93 \| 32.3 \| \| 0.40 \| 0.04 \| 2.63 \| 0.81 \| -25.92 \| 24.4 \| \| 0.45 \| 0.01 \| 2.88 \| 0.17 \| -26.51 \| 20 \| \| 0.50 \| 0.01 \| 2.37 \| 0.15 \| -26.67 \| 19.2 \| \| **MIN** \| 0.01 \| 2.33 \| 0.15 \| -30.82 \| 19.2 \| \| **MAX** \| 0.32 \| 4.21 \| 8.15 \| -25.92 \| 39.6 \| \| **MED.** \| 0.17 \| 2.73 \| 4.70 \| -26.99 \| 28.3 \| \| **AVE** \| 0.16 \| 3.03 \| 4.11 \| -27.51 \| 28.3 \| \| **SD** \| 0.11 \| 0.67 \| 2.96 \| 1.38 \| 6.0 \| | \| **Depth** \| **N_tot_[%]** \| **δ^15^N[‰]** \| **C_org_[%]** \| **δ^13^C[‰]** \| **C/N** \| \| --- \| --- \| --- \| --- \| --- \| --- \| \| Retention tank No 5 ouflow (R 5 OUT) \| \| \| \| \| \| \| 0.05 \| 0.52 \| 4.4 \| 7.58 \| -27.45 \| 16.9 \| \| 0.10 \| 0.14 \| 3.05 \| 2.62 \| -30.27 \| 22.2 \| \| 0.15 \| 0.1 \| 3.2 \| 1.75 \| -36.61 \| 21.0 \| \| 0.20 \| 0.08 \| 2.71 \| 1.43 \| -26.49 \| 20.4 \| \| 0.25 \| 0.08 \| 2.97 \| 1.36 \| -26.13 \| 20.4 \| \| 0.30 \| 0.13 \| 3.45 \| 2.72 \| -26.75 \| 24.4 \| \| 0.35 \| 0.17 \| 3.88 \| 2.87 \| -26.65 \| 20.1 \| \| 0.40 \| 0.22 \| 4.23 \| 4.01 \| -26.3 \| 21.3 \| \| 0.45 \| 0.21 \| 3.8 \| 3.74 \| -25.78 \| 21.1 \| \| **MIN** \| 0.08 \| 2.71 \| 1.36 \| -36.61 \| 16.9 \| \| **MAX** \| 0.52 \| 4.40 \| 7.58 \| -25.78 \| 24.4 \| \| **MED.** \| 0.14 \| 3.45 \| 2.72 \| -26.65 \| 21.0 \| \| **AVE** \| 0.18 \| 3.52 \| 3.12 \| -28.04 \| 20.9 \| \| **SD** \| 0.13 \| 0.56 \| 1.81 \| 3,28 \| 1.9 \| |
|  |  |
| \| **Depth** \| **N_tot_[%]** \| **δ^15^N[‰]** \| **C_org_[%]** \| **δ^13^C[‰]** \| **C/N** \| \| --- \| --- \| --- \| --- \| --- \| --- \| \| Retention tank No 3 inflow (R 3 IN) \| \| \| \| \| \| \| 0.05 \| 0.02 \| 3.75 \| 0.4 \| -26.64 \| 28.9 \| \| 0.10 \| 0.01 \| 3.75 \| 0.18 \| -26.5 \| 28.5 \| \| 0.15 \| 0.01 \| 3.85 \| 0.17 \| -26.4 \| 23.5 \| \| 0.20 \| 0.05 \| 3.85 \| 0.96 \| -27.06 \| 24.8 \| \| 0.25 \| 0.01 \| 2.18 \| 0.44 \| -34.6 \| 37.3 \| \| 0.30 \| 0.01 \| 2.18 \| 0.09 \| -28.05 \| 12.5 \| \| 0.35 \| 0.01 \| 2.37 \| 0.15 \| -30.4 \| 22.9 \| \| 0.40 \| 0.03 \| 1.32 \| 0.88 \| -28.31 \| 33.5 \| \| 0.45 \| 0.03 \| 7.13 \| 0.74 \| -26.67 \| 29.2 \| \| 0.50 \| 0.08 \| 3.6 \| 1.6 \| -26.89 \| 24.0 \| \| 0.55 \| 0.10 \| 3.99 \| 2.57 \| -29.09 \| 31.2 \| \| **MIN** \| 0.01 \| 1.32 \| 0.09 \| -34.60 \| 12.5 \| \| **MAX** \| 0.10 \| 7.13 \| 2.57 \| -26.40 \| 37.3 \| \| **MED.** \| 0.02 \| 3.75 \| 0.44 \| -27.06 \| 28.5 \| \| **AVE** \| 0.03 \| 3.45 \| 0.74 \| -28.24 \| 26.9 \| \| **SD** \| 0.03 \| 1.46 \| 0.73 \| 2.34 \| 6.2 \| | \| **Depth** \| **N_tot_[%]** \| **δ^15^N[‰]** \| **C_org_[%]** \| **δ^13^C[‰]** \| **C/N** \| \| --- \| --- \| --- \| --- \| --- \| --- \| \| Retention tank No 3 outflow (R 3 OUT) \| \| \| \| \| \| \| 0.05 \| 0.19 \| 3.99 \| 4.06 \| -28.47 \| 25.1 \| \| 0.10 \| 0.2 \| 2.47 \| 3.94 \| -26.36 \| 23.6 \| \| 0.15 \| 0.19 \| 3.43 \| 3.31 \| -27.54 \| 20.5 \| \| 0.20 \| 0.16 \| 2.04 \| 2.73 \| -28.78 \| 19.8 \| \| 0.25 \| 0.15 \| 2.17 \| 2.68 \| -27.48 \| 20.6 \| \| 0.30 \| 0.1 \| 4.32 \| 2.08 \| -30.91 \| 24.9 \| \| 0.35 \| 0.13 \| 1.07 \| 2.53 \| -29.26 \| 21.9 \| \| 0.40 \| 0.12 \| 4.44 \| 2.47 \| -29.92 \| 25.0 \| \| 0.45 \| 0.12 \| 4.45 \| 2.96 \| -29.48 \| 28.0 \| \| 0.50 \| 0.09 \| 4.83 \| 1.89 \| -38 \| 24.3 \| \| 0.55 \| 0.13 \| 2.43 \| 2.23 \| -26.73 \| 19.8 \| \| 0.60 \| 0.22 \| 3.3 \| 2.75 \| -26.74 \| 14.3 \| \| 0.65 \| 0.28 \| 3.6 \| 3.72 \| -28.87 \| 15.5 \| \| 0.70 \| 0.32 \| 3.06 \| 4.19 \| -28.91 \| 15.5 \| \| **MIN** \| 0.09 \| 1.07 \| 1.89 \| -38.00 \| 14.3 \| \| **MAX** \| 0.32 \| 4.83 \| 4.19 \| -26.36 \| 28.0 \| \| **MED.** \| 0.16 \| 3.37 \| 2.74 \| -28.82 \| 21.2 \| \| **AVE** \| 0.17 \| 3.26 \| 2.97 \| -29.10 \| 21.3 \| \| **SD** \| 0.06 \| 1.06 \| 0.73 \| 2.77 \| 4.0 \| |
|  |  |
| \| **Depth** \| **N_tot_[%]** \| **δ^15^N[‰]** \| **C_org_[%]** \| **δ^13^C[‰]** \| **C/N** \| \| --- \| --- \| --- \| --- \| --- \| --- \| \| Retention tank No 1 inflow (R 1 IN) \| \| \| \| \| \| \| 0.05 \| 0.14 \| 1.14 \| 2.55 \| -27.06 \| 21.6 \| \| 0.10 \| 0.11 \| 3.32 \| 2.25 \| -28.7 \| 24.3 \| \| 0.15 \| 0.07 \| 2.64 \| 1.53 \| -28.04 \| 25.4 \| \| 0.20 \| 0.06 \| 2.68 \| 1.07 \| -31.03 \| 21.6 \| \| 0.25 \| 0.06 \| 2.99 \| 1.02 \| -28.06 \| 21.1 \| \| 0.30 \| 0.09 \| 2.42 \| 1.44 \| -28.13 \| 17.8 \| \| 0.35 \| 0.11 \| 2.9 \| 1.92 \| -29.24 \| 19.9 \| \| 0.40 \| 0.15 \| 2.72 \| 2.6 \| -27.5 \| 20.3 \| \| 0.45 \| 0.12 \| 1.24 \| 2.08 \| -32.05 \| 20.2 \| \| 0.50 \| 0.19 \| 1.48 \| 3.1 \| -31.66 \| 19.5 \| \| 0.55 \| 0.06 \| 1.38 \| 0.96 \| -31.34 \| 18.6 \| \| 0.60 \| 0.06 \| 2.57 \| 0.96 \| -26.81 \| 17.8 \| \| **MIN** \| 0.06 \| 1.14 \| 0.96 \| -32.05 \| 17.8 \| \| **MAX** \| 0.19 \| 3.32 \| 3.10 \| -26.81 \| 25.4 \| \| **MED.** \| 0.10 \| 2.61 \| 1.73 \| -28.42 \| 20.2 \| \| **AVE** \| 0.10 \| 2.29 \| 1.79 \| -29.13 \| 20.7 \| \| **SD** \| 0.04 \| 0.73 \| 0.70 \| 1.81 \| 2.3 \| | \| **Depth** \| **N_tot_[%]** \| **δ^15^N[‰]** \| **C_org_[%]** \| **δ^13^C[‰]** \| **C/N** \| \| --- \| --- \| --- \| --- \| --- \| --- \| \| Retention tank No 1 outflow (R 1 OUT) \| \| \| \| \| \| \| 0.05 \| 0.06 \| 5.18 \| 0.79 \| -27.08 \| 16.4 \| \| 0.10 \| 0.09 \| 2.51 \| 1.17 \| -27.21 \| 15.2 \| \| 0.15 \| 0.17 \| 2.33 \| 2.31 \| -27.11 \| 16.2 \| \| 0.20 \| 0.2 \| 2.58 \| 3.97 \| -27.02 \| 23.2 \| \| 0.25 \| 0.23 \| 2.97 \| 3.41 \| -26.93 \| 17.7 \| \| 0.30 \| 0.19 \| 3.62 \| 2.78 \| -26.27 \| 17.2 \| \| 0.35 \| 0.13 \| 2.65 \| 2.18 \| -26.28 \| 19.1 \| \| 0.40 \| 0.13 \| 2.19 \| 2.32 \| -26.4 \| 20.7 \| \| 0.45 \| 0.06 \| 3.26 \| 1.3 \| -26.6 \| 25.0 \| \| 0.50 \| 0.08 \| 2.4 \| 1.76 \| -26.94 \| 25.4 \| \| 0.55 \| 0.12 \| 1.32 \| 2.56 \| -28.02 \| 25.2 \| \| 0.60 \| 0.18 \| 1.58 \| 2.73 \| -29.6 \| 18.2 \| \| **MIN** \| 0.06 \| 1.32 \| 0.79 \| -29.60 \| 15.2 \| \| **MAX** \| 0.23 \| 5.18 \| 3.97 \| -26.27 \| 25.4 \| \| **MED.** \| 0.13 \| 2.54 \| 2.32 \| -26.98 \| 18.7 \| \| **AVE** \| 0.14 \| 2.72 \| 2.27 \| -27.12 \| 19.9 \| \| **SD** \| 0.05 \| 0.96 \| 0.88 \| 0.88 \| 3.7 \| |
